# Drug‐facilitated crime: A review of findings between 2019 and 2023

**DOI:** 10.1111/1556-4029.70151

**Published:** 2025-08-12

**Authors:** Meaghan R. Hessler, Sherri L. Kacinko, Barry K. Logan

**Affiliations:** ^1^ Toxicology NMS Labs Horsham Pennsylvania USA

**Keywords:** DFC, DFSA, drug‐facilitated crime, drug‐facilitated sexual assault, drug trends, forensic toxicology

## Abstract

Drug‐facilitated crime (DFC) is a criminal act (e.g., assault, robbery, or sexual assault) in which the perpetrator uses drugs to impair the victim's ability to resist, remember, or recognize the crime being committed. Ethanol is commonly implicated in DFC casework, but limited data are published on other substances currently implicated in these crimes. DFC cases submitted to a large forensic reference laboratory between 2019 and 2023 were analyzed. Analytes and combinations were evaluated based on effect class. In total, 2371 blood samples and 5,041 urine samples were tested for common alcohols, GHB, and a variety of illicit and prescription medications. The most prevalent drug classes were cannabinoids (delta‐9‐THC/delta‐9‐THCCOOH), ethanol, and stimulants, with delta‐9 THC/metabolites being most prevalent in both matrices, followed closely by ethanol. The stimulant drug class's most prevalent analytes include methamphetamine, cocaine/cocaine metabolites, and amphetamine. Gamma‐hydroxybutyric acid (GHB) and flunitrazepam were infrequently detected. Polydrug combination data showed similar trends to when substances were identified alone, specifically that ethanol, cannabinoids, or a stimulant were often found in combination. Sedating substances were more prevalent than stimulating substances, specifically benzodiazepines and antihistamines. Polydrug data support urine collection in DFC cases, as they identified analytes in 22% of cases with negative blood toxicology. Recent literature notes novel psychoactive substances, including designer benzodiazepines, being detected in DFC cases globally, and recommends their inclusion in comprehensive DFC scopes. It is imperative that the appropriate matrix, scope, and limitations be evaluated to accurately determine trends, and scopes are continuously updated to capture the ever‐changing drug market.

**Highlights**

- DFC is underreported; thus, drug positivity in these cases is underestimated.
- Public perceptions of drugs in DFC cases often differ from the substances detected in cases.
- Most positive DFC cases involve multiple substances, stressing the need for comprehensive testing.
- Drug trends in DFC cases, including by age, must be understood to better aid investigations.
- Paired matrices support urine as the best matrix for DFC cases.

## 1 INTRODUCTION

Drug‐facilitated crime (DFC) occurs when an individual uses a substance to alter a victim's mind, thus preventing them from resisting, remembering, consenting, or recognizing that a crime is being committed against them. This often involves administering drugs without the victim's knowledge or consent, leading to reduced awareness, memory impairment, or physical incapacitation. Common types of drug‐facilitated crimes include sexual assault, robbery, and kidnapping. Sexual activity when consent is not freely provided or obtained is defined as sexual violence and is common across the United States and internationally. As of June 2022, the Centers for Disease Control and Prevention (CDC) reports over 50% of women and almost 33% of men have experienced some form of physical sexual violence during their lifetime [1]. Rape specifically refers to nonconsensual sexual intercourse, typically involving penetration of the vagina, anus, or mouth by the perpetrator's body part or an object, without the victim's consent. Twenty‐five percent of women and 4% of men have experienced some form of rape in their lifetime, including completed or attempted incidents [1]. In this study, c. 1 in 20 women became pregnant after rape or sexual coercion during their lifetime, and over 3 million people have experienced rape‐related pregnancy [2]. Marginalized communities including those who identify as transgender, genderqueer, or a nonconforming gender, as well as Native Americans, experience higher risks of sexual violence and sexual assault or rape [3]. Sexual assault is a broader term that encompasses a wide range of nonconsensual sexual activities, not limited to rape. It can include unwanted sexual touching, fondling, groping, forced kissing, and any other nonconsensual sexual contact.

Consent limitations can include age, mental illness, or mental deficiency, as well as intoxication or unconsciousness. Lack of consent can be due to force, threat, coercion, or the victim's incapacitation. DFC is underreported for a variety of reasons, including the nature of the crime, the victim's negative feelings, shame, or guilt surrounding the incident, discomfort in disclosing the event, poor or no recollection of the actual event resulting from drug or alcohol‐related memory loss, including a lack of recollection by the victim of whether or not they gave consent, or threats from their assailant about reporting the event [1, 4, 5]. Once consent has been given, it can also be withdrawn at any time [6]. A lack of consent has also been used as a defense in falsified sexual assault reports postincident [7].

Two categorizations of DFC have been identified: proactive DFC and opportunistic DFC. Proactive DFC is when a victim unknowingly ingests or is forced or encouraged to ingest a substance(s) by the perpetrator(s) to compromise their consent and/or awareness, which could lead to incapacitation. Opportunistic DFC is when a victim knowingly or spontaneously ingests a substance(s) that can compromise their consent and/or awareness and is subsequently taken advantage of by a perpetrator(s). Irrespective of whether an individual knowingly or unknowingly ingests a drug and becomes a victim of a crime, both situations would still constitute a DFC [8].

An authoritative standard with contemporary consensus guidance has recently been approved by the American Academy of Forensic Sciences Standards Board (ASB) as standard ASB 121 [9] and adopted by the Society of Forensic Toxicologists (SOFT), who developed their own recommendations [10]. The suggested scope of analysis in the standard, which is provided for urine only, includes a variety of impairing licit and illicit substances as well as recommended reporting limit thresholds to attempt to differentiate between endogenous versus exogenous administration of select substances, including gamma‐hydroxybutyrate (GHB) [11, 12]. The standard was established to address the specific circumstance of detecting a substance in a urine sample following a single oral dose, with the further assumption that the detection window has not been exceeded because of drug metabolism and elimination. This may not be the fact pattern in every case; however, investigators should be encouraged to consult an expert when recommending collection, storage, and scope of analysis in each case. This standard is expected to be updated on a 3–4‐year cycle, making interim data—such as that presented here—timelier and more pertinent. Regular updates on drug positivity in DFC cases can be a critical resource for toxicologists, investigators, sexual assault nurse examiners (SANEs), sexual health advisors, and counselors on best practices when a potential victim reports their assault.

## 2 MATERIALS AND METHODS

Data were extracted from NMS Labs (Horsham, PA) laboratory information management system (Horizon® LIMS, Clinisys, Raleigh, NC) for all cases with blood and/or urine submitted for DFC testing between January 1, 2019 and December 31, 2023. Cases that had testing beyond the DFC panel were excluded from the analysis. DFC testing consisted of an immunoassay screen and a liquid chromatography time‐of‐flight mass spectrometry screen to cover commonly encountered drugs, screening by gas chromatography–mass spectrometry for GHB, and analysis by headspace gas chromatography for alcohols. Scope and sensitivity were derived from ASB standard 121 [9]. All methods employed were fully validated. All positive findings were confirmed by appropriate validated confirmation techniques. The scope of testing in blood and urine is included in Table 1 as well as updates made to the scope during the analysis period to better align with the ASB required scope of analysis. Chlorophenylpiperazine (mCPP), norchlorcyclizine, and zolpidem carboxylic acid were not included in the scope for the 5‐year analysis period. Analytes are grouped into parent/metabolite pairs to prevent duplicate positivity counts. In blood, heroin‐positive cases were defined as any cases with both morphine and 6‐MAM detected in any matrix; morphine cases are defined as cases positive for morphine without 6‐MAM. As amphetamine is most commonly present resulting from methamphetamine metabolism, cases containing both methamphetamine and amphetamine, or just methamphetamine, counted toward methamphetamine positivity only. Cases categorized as “amphetamine positives” contained amphetamine in the absence of methamphetamine. The same rationale was applied to cases containing oxycodone and oxymorphone. Drugs administered for DFCs may be selected for administration based on a desired pharmacological outcome; therefore, each drug was assigned to a pharmacological “effect group” to simplify assessment and positivity based on a drug's constellation of effects, rather than simply comparing individual compounds, many of which were low numbers. The major groups were “sedating” and “stimulating,” while ethanol and cannabinoids were identified separately due to their high prevalence and widespread recreational use. Within the sedating and stimulating effect groups, drug classes were identified for prevalence within these categories.

**TABLE 1:** NMS scope, analyte classifications, effects, screen cutoff concentrations, and confirmation reporting limits as of December 31, 2023 (screen cutoff/confirmation reporting limit)^d^.

Analyte	Analyte/metabolite pair	Classification	Effect group	Urine	Blood
11‐Hydroxy Delta‐9 THC^a^	Delta‐9 THC/Metabolite	Cannabinoid	Cannabinoid	Not in scope	NA^e^/1.0
1‐Hydroxymidazolam^a^	Midazolam/1‐Hydroxymidazolam	Benzodiazepine	Sedating	5.0/5.0	Not in scope
6‐Monoacetylmorphine^a^	Heroin	Opioid	Sedating	2.0/5.0	2.0/1.0
7‐Amino Clonazepam^c^	Clonazepam/7‐Aminoclonazepam	Benzodiazepine	Sedating	5.0/5.0	5.0/5.0
7‐Amino Flunitrazepam^a^	Flunitrazepam/7‐aminoflunitrazepam/Norflunitrazepam	Benzodiazepine	Sedating	5.0/2.0	5.0/2.0
Acetone^a^	Acetone	Ketone	–^f^	5.0/5.0 mg/dL	5.0/5.0 mg/dL
Acetylfentanyl^a^	Fentanyl/Fentanyl Marker	Opioid	Sedating	0.50/1.0	0.50/0.20
Alpha‐Hydroxyalprazolam^b^	Alprazolam/Alpha‐Hydroxyalprazolam	Benzodiazepine	Sedating	10/10	10/5.0
Alprazolam^a^	Alprazolam/Alpha‐Hydroxyalprazolam	Benzodiazepine	Sedating	10/5.0	10/5.0
Amitriptyline^b^	Amitriptyline/Nortriptyline	Antidepressant	Sedating	10/50	10/20
Amphetamine^b^	Amphetamine	Stimulant	Stimulant	10/50	10/5.0
Benzoylecgonine^c^	Cocaine/Cocaine Metabolite	Stimulant	Stimulant	50/50^g^	50/50
Brompheniramine^b^	Brompheniramine	Antihistamine	Sedating	10/20	10/40
Buprenorphine^a^	Buprenorphine/Norbuprenorphine	Opioid	Sedating	1.0/5.0	1.0/0.50
Butalbital^b^	Butalbital	Anticonvulsant, Sedative	Sedating	NA^h^/200	NA^h^/200
Carisoprodol^b^	Carisoprodol/Meprobamate	Muscle Relaxant	Sedating	50/200	50/200
Chlordiazepoxide^a^	Chlordiazepoxide	Benzodiazepine	Sedating	10/20	10/20
Chlorpheniramine^c^	Chlorpheniramine	Antihistamine	Sedating	10/10	10/10
Citalopram/Escitalopram^a^	Citalopram/Escitalopram	Antidepressant	–^f^	10/5.0	10/5.0
Clobazam^a^	Clobazam	Benzodiazepine	Sedating	10/20	10/20
Clonazepam^a^	Clonazepam/7‐Aminoclonazepam	Benzodiazepine	Sedating	Not in scope	5.0/2.0
Clonidine^a^	Clonidine	Antihypertensive	–^f^	1.0/0.50	1.0/0.10
Cocaethylene^a^	Cocaine/Cocaine Metabolite	Stimulant	Stimulant	20/10^i^	20/10
Cocaine^a^	Cocaine/Cocaine Metabolite	Stimulant	Stimulant	20/10^i^	20/10
Codeine^b^	Codeine	Opioid	Sedating	10/25^j^	10/5.0
Cyclobenzaprine^c^	Cyclobenzaprine	Muscle Relaxant	Sedating	10/1.0	10/1.0
Delta‐9 Carboxy THC^c^	Delta‐9 Carboxy THC—Total	Cannabinoid	Cannabinoid	20^k^/5.0	10/5.0
Delta‐9 THC^a^	Delta‐9 THC/Metabolite	Cannabinoid	Cannabinoid	Not in scope	NA^e^/0.50
Desalkylflurazepam^a^	Flurazepam/Desalkylflurazepam/Hydroxyethylflurazepam	Benzodiazepine	Sedating	10/5.0	10/5.0
Desipramine^b^	Desipramine	Antidepressant	Sedating	10/100	10/20
Desmethyldoxepin^a^	Doxepin/Desmethyldoxepin	Antidepressant	Sedating	10/50	10/20
Desmethylsertraline^a^	Sertraline/Desmethylsertraline	Antidepressant	–^f^	Not in scope	10/20
Dextro/Levo Methorphan^c^	D‐/L‐Methorphan/Metabolites	Antitussive/Narcotic Analgesic	Sedating	10/10	10/5.0
Dextrorphan/Levorphanol^a^	D‐/L‐Methorphan/Metabolites	Antitussive/Narcotic Analgesic	Sedating	100/100	100/2.0
Diazepam^a^	Diazepam/Diazepam Metabolite	Benzodiazepine	Sedating	10/20	10/20
Dihydrocodeine/Hydrocodol^a^	Hydrocodone/Dihydrocodeine/Hydrocodol	Opioid	Sedating	10/25^j^	10/5.0
Diphenhydramine^b^	Diphenhydramine	Antihistamine	Sedating	50/50	50/50
Doxepin^a^	Doxepin/Desmethyldoxepin	Antidepressant	Sedating	10/50	10/20
Doxylamine^b^	Doxylamine	Antihistamine	Sedating	10/50	10/100
EDDP^a^	Methadone/EDDP	Opioid	Sedating	10/20^i^	10/20
Estazolam^a^	Estazolam	Benzodiazepine	Sedating	10/5.0	10/5.0
Eszopiclone/Zopiclone^c^	Eszopiclone/Zopiclone	Sleep Aid	Sedating	2.0/2.0	2.0/2.0
Ethanol^c^	Ethanol	Ethanol	Ethanol	10/10 mg/dL	10/10 mg/dL
Fentanyl^c^	Fentanyl/Fentanyl Marker	Opioid	Sedating	1.0/1.0	1.0/0.20
Flunitrazepam^a^	Flunitrazepam/7‐aminoflunitrazepam/Norflunitrazepam	Benzodiazepine	Sedating	5.0/2.0	5.0/2.0
Fluoxetine^a^	Fluoxetine/Norfluoxetine	Antidepressant	–^f^	10/10^l^	10/20
Flurazepam^a^	Flurazepam/Desalkylflurazepam/Hydroxyethylflurazepam	Benzodiazepine	Sedating	Not in scope	NA^m^/2.0
Gamma‐Hydroxybutyric Acid^c^	Gamma‐Hydroxybutyric Acid	Hypnotic, Sedative	Sedating	5.0/5.0 mcg/mL	5.0/5.0 mcg/mL
Hydrocodone^b^	Hydrocodone/Dihydrocodeine/Hydrocodol	Opioid	Sedating	10/25^j^	10/5.0
Hydromorphone^c^	Hydromorphone	Opioid	Sedating	2.0/5.0^n^	2.0/1.0
Hydroxyethylflurazepam^a^	Flurazepam/Desal ylflurazepam/Hydroxyethylflurazepam	Benzodiazepine	Sedating	10/5.0	10/5.0
Hydroxytriazolam^a^	Triazolam/Hydroxytriazolam	Benzodiazepine	Sedating	5.0/5.0	5.0/5.0
Imipramine^b^	Imipramine	Antidepressant	Sedating	10/100	10/20
Isopropanol^a^	Isopropanol	Alcohol	–^m^	5/5 mg/dL	5.0/5.0
Ketamine^a^	Ketamine/Norketamine	Hypnotic, Sedative	Sedating	20/20^o^	20/40
Lidocaine^a^	Lidocaine/MEGX	Anesthetic (Local)	–^m^	200/500	200/500
Lorazepam^b^	Lorazepam	Benzodiazepine	Sedating	5/10	5.0/5.0
MDA^b^	MDMA/MDA	Stimulant	Stimulant	10/50	10/5.0
MDMA^b^	MDMA/MDA	Stimulant	Stimulant	10/50	10/5.0
Meperidine^a^	Meperidine/Normeperidine	Opioid	Sedating	10/20	10/40
Meprobamate^b^	Carisoprodol/Meprobamate	Muscle Relaxant	Sedating	50/1000	50/1000
Methadone^a^	Methadone/EDDP	Opioid	Sedating	10/20^i^	10/20
Methamphetamine^b^	Methamphetamine	Stimulant	Stimulant	10/50	10/5.0
Methanol^a^	Methanol	Alcohol	–^m^	10/10^p^ mg/dL	10/10^p^
Midazolam^a^	Midazolam/1‐Hydroxymidazolam	Benzodiazepine	Sedating	Not in scope	5.0/5.0
Monoethylglycinexylidide (MEGX)^a^	Lidocaine/MEGX	Anesthetic (Local)	–^m^	200/500	200/500
Morphine^b^	Morphine	Opioid	Sedating	10/25^j^	10/5.0
Norbuprenorphine^a^	Buprenorphine/Norbuprenorphine	Opioid	Sedating	2.0/5.0	2.0/0.50
Nordiazepam^b^	Diazepam/Diazepam Metabolite	Benzodiazepine	Sedating	10/20	10/20
Norfentanyl^c^	Fentanyl/Fentanyl Marker	Opioid	Sedating	1.0/1.0	1.0/0.40
Norflunitrazepam^a^	Flunitrazepam/7‐aminoflunitrazepam/Norflunitrazepam	Benzodiazepine	Sedating	20/2.0	20/2.0
Norfluoxetine^a^	Fluoxetine/Norfluoxetine	Antidepressant	–^m^	10/10^l^	10/20
Norketamine^b^	Ketamine/Norketamine	Hypnotic, Sedative	Sedating	20/20^q^	20/40
Normeperidine^a^	Meperidine/Normeperidine	Opioid	Sedating	10/20	10/20
Norpropoxyphene^a^	Propoxyphene/Norpropoxyphene	Opioid	Sedating	10/100	10/100
Nortriptyline^b^	Amitriptyline/Nortriptyline	Antidepressant	Sedating	10/50	10/20
O‐Desmethyltramadol^a^	Tramadol/O‐desmehtyltramadol	Opioid	Sedating	10/10^r^	10/20
Oxazepam^b^	Diazepam/Diazepam Metabolite	Benzodiazepine	Sedating	10/20	10/20
Oxycodone^b^	Oxycodone	Opioid	Sedating	10/25^j^	10/5.0
Oxymorphone^c^	Oxymorphone	Opioid	Sedating	2.0/5.0^n^	2.0/1.0
Paroxetine^a^	Paroxetine	Antidepressant	–^m^	10/10	10/10
Pentobarbital^b^	Pentobarbital	Anticonvulsant, Sedative	Sedating	NA^h^/200	NA^h^/200
Phencyclidine^a^	Phencyclidine	Dissociative	Sedating	5.0/5.0	5.0/5.0
Phenobarbital^b^	Phenobarbital	Anticonvulsant, Sedative	Sedating	NA^h^/500	NA^h^/500
Phenytoin^a^	Phenytoin	Anticonvulsant, Antiepileptic	Sedating	500/500	500/500
Propoxyphene^a^	Propoxyphene/Norpropoxyphene	Opioid	Sedating	10/100	10/100
Scopolamine^a^	Scopolamine	Anticholinergic	Sedating	5.0/0.40	10/0.40
Secobarbital^a^	Secobarbital	Anticonvulsant, Sedative	Sedating	300/200	40/200
Sertraline^a^	Sertraline/Desmethylsertraline	Antidepressant	–^m^	10/10^s^	10/10
Temazepam^b^	Diazepam/Diazepam Metabolite	Benzodiazepine	Sedating	10/20	10/20
Tetrahydrozoline^a^	Tetrahydrozoline	Ocular Vasoconstrictor	–^m^	0.050/0.050	0.050/0.10
Tramadol^c^	Tramadol/O‐desmethyltramadol	Opioid	Sedating	10/10^r^	10/20
Triazolam^a^	Triazolam/Hydroxytriazolam	Benzodiazepine	Sedating	Not in scope	5.0/2.0
Zaleplon^a^	Zaleplon	Sleep Aid	Sedating	5.0/4.0	5.0/4.0
Ziprasidone^a^	Ziprasidone	Antipsychotic	Sedating	Not in scope	2.0/2.0
Zolpidem^a^	Zolpidem	Sleep Aid	Sedating	10/4.0	10/4.0

^a^ Not included in ASB recommendations.

^b^ Included in ASB recommendations. Does meet required sensitivity.

^c^ Included in ASB recommendations. Meets or exceeds required sensitivity.

^d^ All concentrations units are ng/mL unless otherwise noted.

^e^ Immunoassay targets Delta‐9 Carboxy THC.

^f^ These drugs are not known to have a significant sedating/stimulatory effect.

^g^ Reporting limit changed from 150 to 15 ng/mL on May 18, 2020 and from 15 to 50 ng/mL on April 17, 2023.

^h^ Immunoassay screen targets secobarbital.

^i^ Reporting limit changed from 200 to 20 ng/mL on May 18, 2020 and from 20 to 10 ng/mL on April 17, 2023.

^j^ Reporting limit changed from 250 to 25 ng/mL on June 7, 2021.

^k^ Screen cutoff changed from 50 to 20 ng/mL on November 2, 2023.

^l^ Reporting limit changed from 20 to 10 ng/mL on April 30, 2020.

^m^ Flurazepam not included in screen but included in confirmation panel. If a sample screened positive for another analyte in the confirmation panel and flurazepam was present, it would produce a positive result. No flurazepam positives were reported.

^n^ Reporting limit changed from 50 to 5 ng/mL on June 7, 2021.

^o^ Reporting limit changed from 200 to 20 ng/mL on April 30, 2020.

^p^ Screen cutoff and reporting limit changed from 5 to 10 ng/mL on August 15, 2022.

^q^ Reporting limit changed from 100 to 20 ng/mL on April 30, 2020.

^r^ Reporting limit changed from 100 to 10 ng/mL on April 30, 2020.

^s^ Reporting limit changed from 1 to 10 ng/mL on September 11, 2023.

Demographics, including sex and age, were evaluated. When age was provided, patients were grouped into the following age groups: child (0.5–12 years), teen (13–20 years), adults (21–59 years), and seniors (≥60 years). Twenty‐year‐olds were included in the teen category to account for the legal drinking age for ethanol.

The prevalence of individual analytes and combinations by drug class was examined along with trends in quantitative ethanol results. GHB and creatinine‐corrected GHB concentrations were evaluated at 5 and 10 mcg/mL and 9.6 mcg/g creatinine, respectively.

## 3 RESULTS

Urine and blood underwent comprehensive testing (Table 1). Confirmation reporting limits met or exceeded the ASB required minimum sensitivity in urine unless otherwise noted. During the study period, there were minor scope changes, including removal of amobarbital, butabarbital, and methedrone from the scope, and minor screening cutoff and confirmation reporting limit changes as indicated. There were no positive results for the removed analytes prior to their removal.

From 2019 to 2023, 6051 cases were analyzed under the DFC scope of testing, including 2371 blood samples and 5041 urine samples. Only urine was tested in 3680 cases, while 1361 cases had both urine and blood DFC panels performed. Percent positivity was evaluated by year by matrix. Overall, 65% of blood samples and 80% of urine samples had at least one positive finding with consistent positivity between matrices across all 5 years (Table 2). For both blood and urine, positive samples most commonly contained fewer than three findings, and c. 7% of urine samples and 1% of blood samples had five or more findings.

**TABLE 2:** Annual and total case volume and positivity by matrix.

Matrix	Year	Number tested	Number positive	% positive
Blood	2019	631	430	68.1
	2020	523	355	67.9
	2021	326	199	61.0
	2022	489	310	63.4
	2023	402	253	62.9
	Total	2371	1547	65.2
Urine	2019	893	720	80.6
	2020	1041	814	78.2
	2021	780	620	79.5
	2022	1170	964	82.4
	2023	1157	921	79.6
	Total	5041	4039	80.1

### 3.1 Demographics

Of the 6051 total cases submitted, 3268 were female, 360 were male, and 2423 did not have sex provided. Excluding unknown sex cases, female and male cases account for 90.1% and 9.9% of cases, respectively. Seventy‐nine percent of female and 71% of male cases were positive for at least one analyte. Age was not provided for c. 47% of cases (*n* = 2836). For cases that did have age provided, adults were the largest age group (*n* = 2107, 66%), followed by teens (*n* = 890, 28%), then children (*n* = 132, 4.1%), and seniors (*n* = 59, 1.8%) (Table 3). Adults had the highest positivity at 83%, followed by teens (75%), then seniors (68%), and finally children (28%). Adults also had the highest percent positive for urine cases at 83%.

**TABLE 3:** Case submissions and positivity by age group.

Age group	Matrix	Number tested	Number positive	Percent positive (%)
0.5–12	Blood	13	2	15
	Urine	112	29	26
13–20	Blood	199	126	63
	Urine	798	619	78
21–59	Blood	408	333	82
	Urine	1892	1572	83
≥60	Blood	15	11	73
	Urine	48	33	69

### 3.2 Blood and urine cases

**FIGURE 1:**
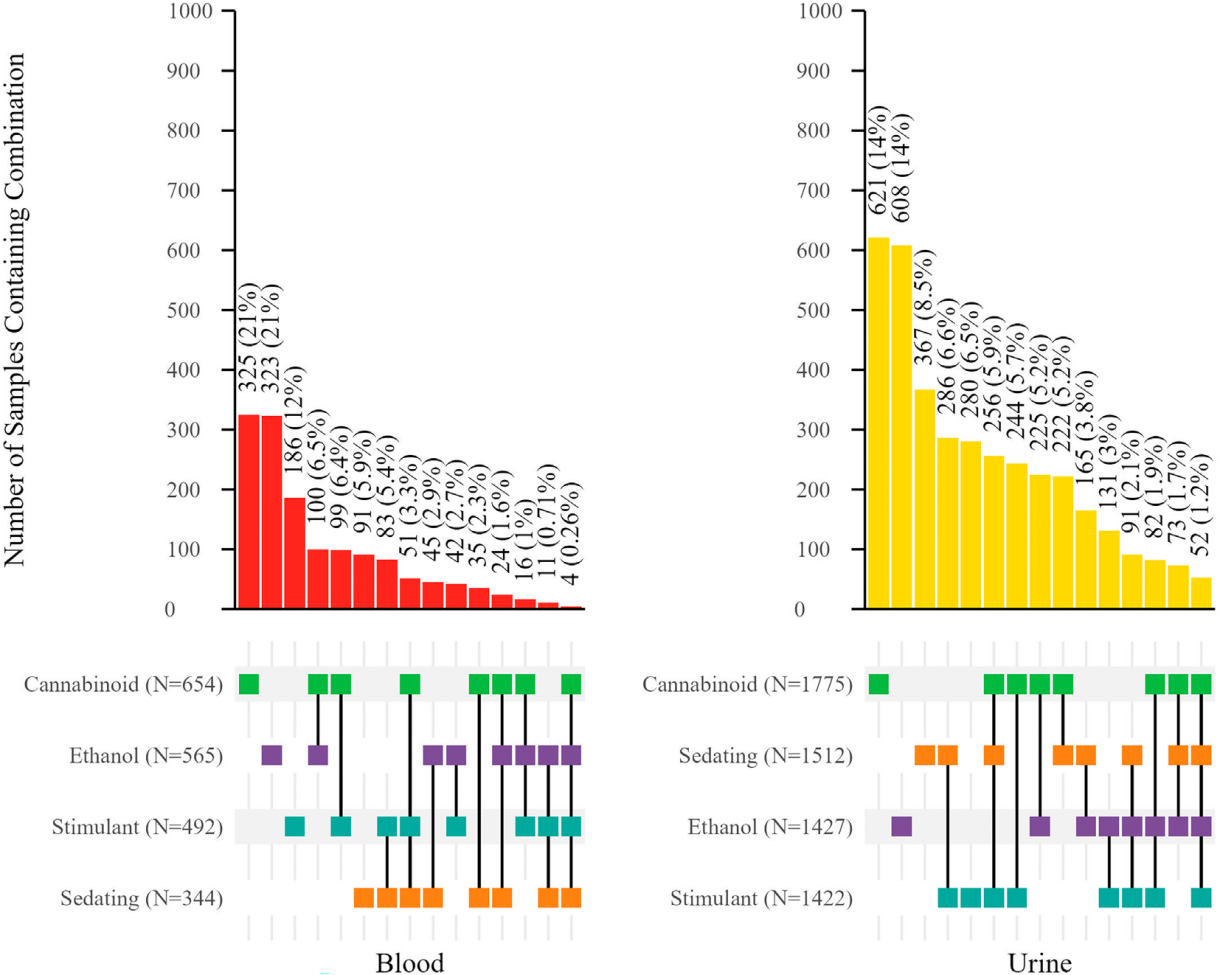
Combinations of drug effect groups seen in blood and urine specimens in cases (*N* = 1361) where both matrices were tested. For each group, the total number of detections in each matrix is indicated after the group name. The bar graph represents the number and percent (of total cases tested) that contained the combination indicated by the colored squares below the bars.

Paired samples, where blood and urine samples were submitted for the same case (*n* = 1361), were evaluated for drug positivity comparisons. Figure 1 depicts the prevalence of each analyte in the paired sample and illustrates how often an analyte was detected only in one matrix or in both in a case. Comparisons between analyte detections between matrices are shown in Table 4. In c. 34% of cases, results were consistent between blood and urine matrices. Sixty‐three percent of cases had findings noted in urine samples that were not identified in blood, with the most prevalent analytes being acetone (18%), ethanol (13%), and cocaine/cocaine metabolites (10%). Cases where an analyte was detected in blood but not in urine were substantially less (6.8%), with the most prevalent findings being Delta‐9 THC/metabolites (2.9%), sertraline/desmethylsertraline (1.1%), and ethanol (<1%). In 22% of cases, urine was the only matrix that indicated a positive finding, whereas in 1.8% of cases, blood was the only matrix indicating a positive finding. The results for cases where only one matrix was analyzed focus on urine, with the exception of quantitative ethanol results in blood, as standards and scope recommendations are written explicitly for urine.

**TABLE 4:** Results comparison in 1361 blood and urine in cases where both specimens were tested.

	Number of cases	% of total cases
Same analytes in blood and urine	381	34
One or more analytes found in blood that was not found in urine	77	6.8
One or more analytes found in urine that was not found in blood	713	63
Urine positive, blood negative	255	22
Blood positive, urine negative	20	1.8

### 3.3 Analyte positivity

Positivity for each analyte/metabolite pair is listed in Table 5. Analyte positivity remained consistent year over year, apart from methamphetamine in blood, which decreased from 19% in the first 2 years of the study to c. 10% over the next 3 years.

**TABLE 5:** Number of samples positive (% of total tested cases) for NMS Scope of Analysis.

	Blood						Urine					
Drug/metabolite pair	2019 (*N* = 631)	2020 (*N* = 523)	2021 (*N* = 326)	2022 (*N* = 489)	2023 (*N* = 402)	Total (*N* = 2371)	2019 (*N* = 893)	2020 (*N* = 1041)	2021 (*N* = 780)	2022 (*N* = 1170)	2023 (*N* = 1157)	Total (*N* = 5041)
Acetone	5 (0.79)	10 (1.9)	3 (0.92)	5 (1.0)	4 (1.0)	27 (1.1)	124 (14)	176 (17)	137 (18)	186 (16)	174 (15)	797 (16)
Alprazolam/Alpha‐Hydroxyalprazolam	12 (1.9)	12 (2.3)	5 (1.5)	10 (2.0)	2 (0.5)	41 (1.7)	42 (4.7)	40 (3.8)	27 (3.5)	28 (2.4)	32 (2.8)	169 (3.4)
Amitriptyline/Nortriptyline	4 (0.63)	2 (0.38)	1 (0.31)	–^a^	1 (0.25)	8 (0.34)	5 (0.56)	4 (0.38)	4 (0.51)	4 (0.34)	6 (0.52)	23 (0.46)
Amphetamine	17 (2.7)	11 (2.1)	7 (2.1)	20 (4.1)	16 (4.0)	71 (3.0)	32 (3.6)	48 (4.6)	35 (4.5)	61 (5.2)	55 (4.8)	231 (4.6)
Buprenorphine/Norbuprenorphine	5 (0.79)	2 (0.38)	2 (0.61)	4 (0.82)	3 (0.75)	16 (0.67)	24 (2.7)	24 (2.3)	19 (2.4)	18 (1.5)	18 (1.6)	103 (2.0)
Butalbital	2 (0.32)	–	–	1 (0.2)	–	3 (0.13)	1 (0.11)	2 (0.19)	–	–	1 (0.086)	4 (0.079)
Carisoprodol/Meprobamate	–	–	–	–	1 (0.25)	1 (0.042)	–	2 (0.19)	–	1 (0.085)	‐	3 (0.060)
Chlordiazepoxide	–	2 (0.38)	–	1 (0.20)	–	3 (0.13)	1 (0.11)	4 (0.38)	1 (0.13)	1 (0.085)	1 (0.086)	8 (0.16)
Chlorpheniramine	2 (0.32)	–	–	–	–	2 (0.084)	11 (1.2)	7 (0.67)	5 (0.64)	14 (1.2)	9 (0.78)	46 (0.91)
Citalopram/Escitalopram	26 (4.1)	23 (4.4)	18 (5.5)	15 (3.1)	18 (4.5)	100 (4.2)	47 (5.3)	55 (5.3)	39 (5)	62 (5.3)	72 (6.2)	275 (5.5)
Clobazam	–	–	–	–	–		–	1 (0.096)	‐	1 (0.085)	–	2 (0.040)
Clonazepam/7‐Aminoclonazepam	19 (3.0)	19 (3.6)	7 (2.1)	15 (3.1)	13 (3.2)	73 (3.1)	41 (4.6)	41 (3.9)	29 (3.7)	47 (4)	40 (3.5)	198 (3.9)
Clonidine	3 (0.48)	–	–	8 (1.6)	2 (0.50)	13 (0.55)	11 (1.2)	11 (1.1)	6 (0.77)	26 (2.2)	24 (2.1)	78 (1.5)
Cocaine/Cocaine Metabolite	46 (7.3)	22 (4.2)	13 (4.0)	18 (3.7)	11 (2.7)	110 (4.6)	106 (12)	138 (13)	98 (13)	158 (14)	141 (12)	641 (13)
Codeine	3 (0.48)	–	–	–	1 (0.25)	4 (0.17)	16 (1.8)	15 (1.4)	9 (1.2)	17 (1.5)	11 (0.95)	68 (1.3)
Cyclobenzaprine	2 (0.32)	3 (0.57)	1 (0.31)	–	–	6 (0.25)	14 (1.6)	25 (2.4)	10 (1.3)	18 (1.5)	29 (2.5)	96 (1.9)
D‐/L‐Methorphan/Metabolites	3 (0.48)	–	–	2 (0.41)	–	5 (0.21)	38 (4.3)	27 (2.6)	26 (3.3)	36 (3.1)	36 (3.1)	163 (3.2)
Delta‐9 THC/Metabolite	172 (27)	144 (28)	84 (26)	148 (30)	106 (26)	654 (28)	295 (33)	344 (33)	249 (32)	436 (37)	451 (39)	1775 (35)
Desalkylflurazepam	–	–	–	–	–	–	–	2 (0.19)	–	–	–	2 (0.040)
Desipramine	–	–	–	–	–	–	–	–	–	1 (0.085)	‐	1 (0.020)
Diazepam/	5	13	2	11	5	36	23	35	14	30	36	138
Diazepam Metabolite	(0.79)	(2.5)	(0.61)	(2.2)	(1.2)	(1.5)	(2.6)	(3.4)	(1.8)	(2.6)	(3.1)	(2.7)
Diphenhydramine	13 (2.1)	8 (1.5)	2 (0.61)	2 (0.41)	3 (0.75)	28 (1.2)	71 (8)	67 (6.4)	48 (6.2)	54 (4.6)	83 (7.2)	323 (6.4)
Doxepin/Desmethyldoxepin	–	–	–	1 (0.2)	1 (0.25)	2 (0.084)	–	4 (0.38)	3 (0.38)	1 (0.085)	1 (0.086)	9 (0.18)
Doxylamine	–	–	–	–	–	–	22 (2.5)	21 (2)	12 (1.5)	13 (1.1)	13 (1.1)	81 (1.6)
Estazolam	–	–	–	–	1 (0.25)	1 (0.042)	–	–	–	–	–	–
Eszopiclone/Zopiclone	–	–	–	–	–	–	2 (0.22)	–	1 (0.13)	1 (0.085)	–	4 (0.079)
Ethanol	145 (23)	128 (24)	73 (22)	113 (23)	106 (26)	565 (24)	254 (28)	313 (30)	222 (28)	339 (29)	299 (26)	1427 (28)
Fentanyl/Fentanyl Marker	10 (1.6)	13 (2.5)	3 (0.92)	14 (2.9)	10 (2.5)	50 (2.1)	52 (5.8)	54 (5.2)	40 (5.1)	74 (6.3)	80 (6.9)	300 (6.0)
Flunitrazepam/7‐aminoflunitrazepam/Norflunitrazepam	1 (0.16)	–	–	–	–	1 (0.042)	1 (0.11)	–	–	–	–	1 (0.020)
Fluoxetine/Norfluoxetine	31 (4.9)	27 (5.2)	6 (1.8)	17 (3.5)	14 (3.5)	95 (4.0)	41 (4.6)	54 (5.2)	34 (4.4)	62 (5.3)	60 (5.2)	251 (5.0)
Gamma‐Hydroxybutyric Acid	1 (0.16)	5 (0.96)	1 (0.31)	6 (1.2)	5 (1.2)	18 (0.76)	10 (1.1)	3 (0.29)	7 (0.9)	16 (1.4)	4 (0.35)	40 (0.79)
Heroin	4 (0.63)	1 (0.19)	1 (0.31)	–	–	6 (0.25)	14 (1.6)	12 (1.2)	1 (0.13)	5 (0.43)	1 (0.086)	33 (0.65)
Hydrocodone/Dihydrocodeine/Hydrocodol	2 (0.32)	1 (0.19)	–	2 (0.41)	3 (0.75)	8 (0.34)	6 (0.67)	7 (0.67)	6 (0.77)	9 (0.77)	10 (0.86)	38 (0.75)
Hydromorphone	1 (0.16)	–	1 (0.31)	–	–	2 (0.084)	27 (3.0)	19 (1.8)	9 (1.2)	25 (2.1)	23 (2.0)	103 (2.0)
Isopropanol	–	–	–	–	–	–	–	1 (0.096)	3 (0.38)	2 (0.17)	1 (0.086)	7 (0.14)
Ketamine/Norketamine	–	3 (0.57)	1 (0.31)	1 (0.20)	2 (0.50)	7 (0.30)	7 (0.78)	7 (0.67)	3 (0.38)	3 (0.26)	8 (0.69)	28 (0.56)
Lidocaine/MEGX	1 (0.16)	–	1 (0.31)	–	–	2 (0.084)	14 (1.6)	17 (1.6)	14 (1.8)	16 (1.4)	26 (2.2)	87 (1.7)
Lorazepam	17 (2.7)	21 (4.0)	7 (2.1)	14 (2.9)	17 (4.2)	76 (3.2)	51 (5.7)	55 (5.3)	34 (4.4)	50 (4.3)	59 (5.1)	249 (4.9)
MDMA/MDA	9 (1.4)	3 (0.57)	–	4 (0.82)	1 (0.25)	17 (0.72)	15 (1.7)	9 (0.86)	8 (1.0)	7 (0.60)	5 (0.43)	44 (0.87)
Meperidine/Normeperidine	–	–	–	–	–	–	–	–	–	1 (0.085)	–	1 (0.020)
Methadone/EDDP	5 (0.79)	6 (1.1)	1 (0.31)	3 (0.61)	3 (0.75)	18 (0.76)	11 (1.2)	16 (1.5)	8 (1.0)	11 (0.94)	7 (0.61)	53 (1.1)
Methamphetamine	119 (19)	97 (19)	30 (9.2)	53 (11)	35 (8.7)	334 (14)	174 (19)	166 (16)	100 (13)	156 (13)	170 (15)	766 (15)
Methanol	–	–	–	–	–	–	–	–	–	1 (0.085)	–	1 (0.020)
Midazolam/1‐Hydroxymidazolam	3 (0.48)	5 (0.96)	–	–	2 (0.5)	10 (0.42)	21 (2.4)	19 (1.8)	6 (0.77)	16 (1.4)	11 (0.95)	73 (1.4)
Morphine	1 (0.16)	–	–	–	1 (0.25)	2 (0.084)	25 (2.8)	22 (2.1)	15 (1.9)	25 (2.1)	26 (2.2)	113 (2.2)
Oxycodone	1 (0.16)	3 (0.57)	2 (0.61)	1 (0.2)	4 (1)	11 (0.46)	10 (1.1)	6 (0.58)	13 (1.7)	13 (1.1)	9 (0.78)	51 (1.0)
Oxymorphone	–	–	–	–	–	–	3 (0.34)	6 (0.58)	2 (0.26)	6 (0.51)	6 (0.52)	23 (0.46)
Paroxetine	3 (0.48)	1 (0.19)	–	2 (0.41)	2 (0.50)	8 (0.34)	6 (0.67)	4 (0.38)	3 (0.38)	5 (0.43)	5 (0.43)	23 (0.46)
Phencyclidine	2 (0.32)	1 (0.19)	1 (0.31)	2 (0.41)	–	6 (0.25)	2 (0.22)	11 (1.1)	5 (0.64)	3 (0.26)	7 (0.61)	28 (0.56)
Phenobarbital	2 (0.32)	2 (0.38)	–	1 (0.20)	3 (0.75)	8 (0.34)	2 (0.22)	4 (0.38)	1 (0.13)	3 (0.26)	3 (0.26)	13 (0.26)
Phenytoin	1 (0.16)	–	–	–	–	1 (0.042)	1 (0.11)	–	1 (0.13)	–	–	2 (0.040)
Sertraline/Desmethylsertraline	20 (3.2)	22 (4.2)	11 (3.4)	22 (4.5)	18 (4.5)	93 (3.9)	33 (3.7)	25 (2.4)	26 (3.3)	45 (3.8)	37 (3.2)	166 (3.3)
Tetrahydrozoline	2 (0.32)	1 (0.19)	–	1 (0.2)	1 (0.25)	5 (0.21)	22 (2.5)	24 (2.3)	18 (2.3)	28 (2.4)	23 (2.0)	115 (2.3)
Tramadol/O‐desmethyltramadol	4 (0.63)	1 (0.19)	1 (0.31)	–	2 (0.50)	8 (0.34)	7 (0.78)	17 (1.6)	8 (1.0)	12 (1.0)	11 (0.95)	55 (1.1)
Venlafaxine/O‐Desmethylvenlafaxine	–	–	–	–	–	–	–	–	–	1 (0.085)	–	1 (0.020)
Ziprasidone	1 (0.16)	3 (0.57)	1 (0.31)	5 (1.0)	1 (0.25)	11 (0.46)	–^b^	–	–	–	–	–
Zolpidem	2 (0.32)	2 (0.38)	1 (0.31)	2 (0.41)	2 (0.50)	9 (0.38)	4 (0.45)	3 (0.29)	2 (0.26)	1 (0.085)	2 (0.17)	12 (0.24)

^a^ No positive samples.

^b^ Not included in urine scope.

**FIGURE 2:**
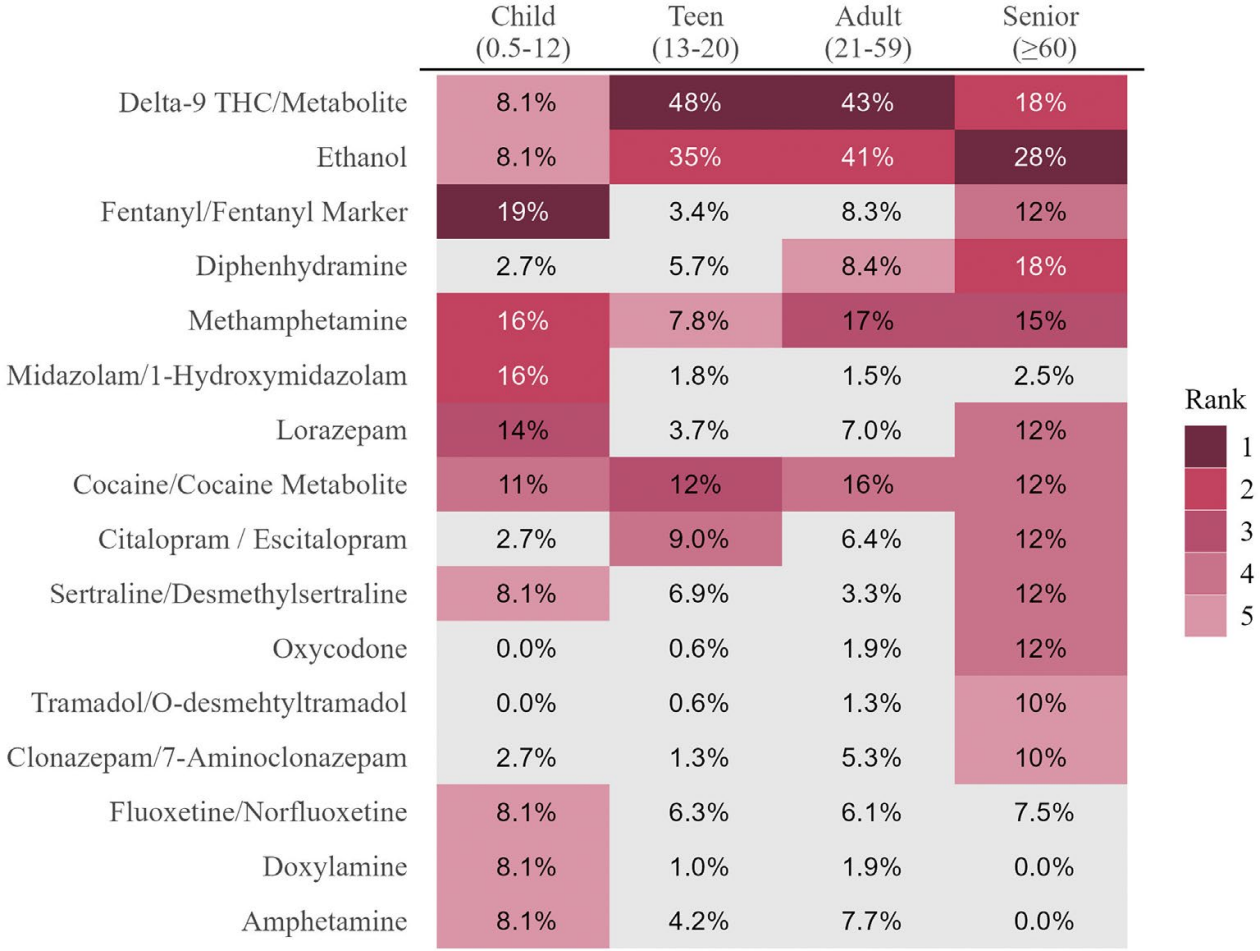
Five most commonly detected analyte(s) in urine by age group (excluding acetone) by percent of total positive cases tested within each age group. Analytes are ranked 1–5 with 1 being the most detected drug. For comparison purposes the percent positive is provided for any analyte that was in the top 5 for any age group (gray boxes). Due to the low number of positive cases for the child and senior age categories, multiple drugs were detected at the same rate so more than five drugs are ranked.

The top five most prevalent drugs by age group are depicted in Figure 2. Due to the low number of samples in the child and senior group, several analytes were detected at the same frequency. Ethanol, delta‐9 THC/metabolite, methamphetamine, and cocaine/metabolite were the only four analytes detected in the top five for all age groups. Ethanol was most prevalent in adults (41%), followed by teens (35%), and was ranked number two for both age groups. While ethanol was ranked first in seniors, percent positivity was 28%, and in children 8.1% and ranked fifth. Cannabinoids were ranked first for teens (48%) and adults (43%) and tied for second in seniors (18%). Minimal frequency was noted in children at 8.1%. Cocaine/metabolites had consistent frequencies across all four groups with c. 12%. Methamphetamine was the only analyte that was in the top five at all ages with similar frequencies, at c. 16%, in children, adults, and seniors; teens had methamphetamine prevalence that was c. 1/2 of the other age groups. Fentanyl/fentanyl marker was identified as the number one finding in children with a frequency of 19%, while not being identified in teens and adults. It was the fourth most prevalent frequency for seniors at 12%.

### 3.4 Ethanol

**FIGURE 3:**
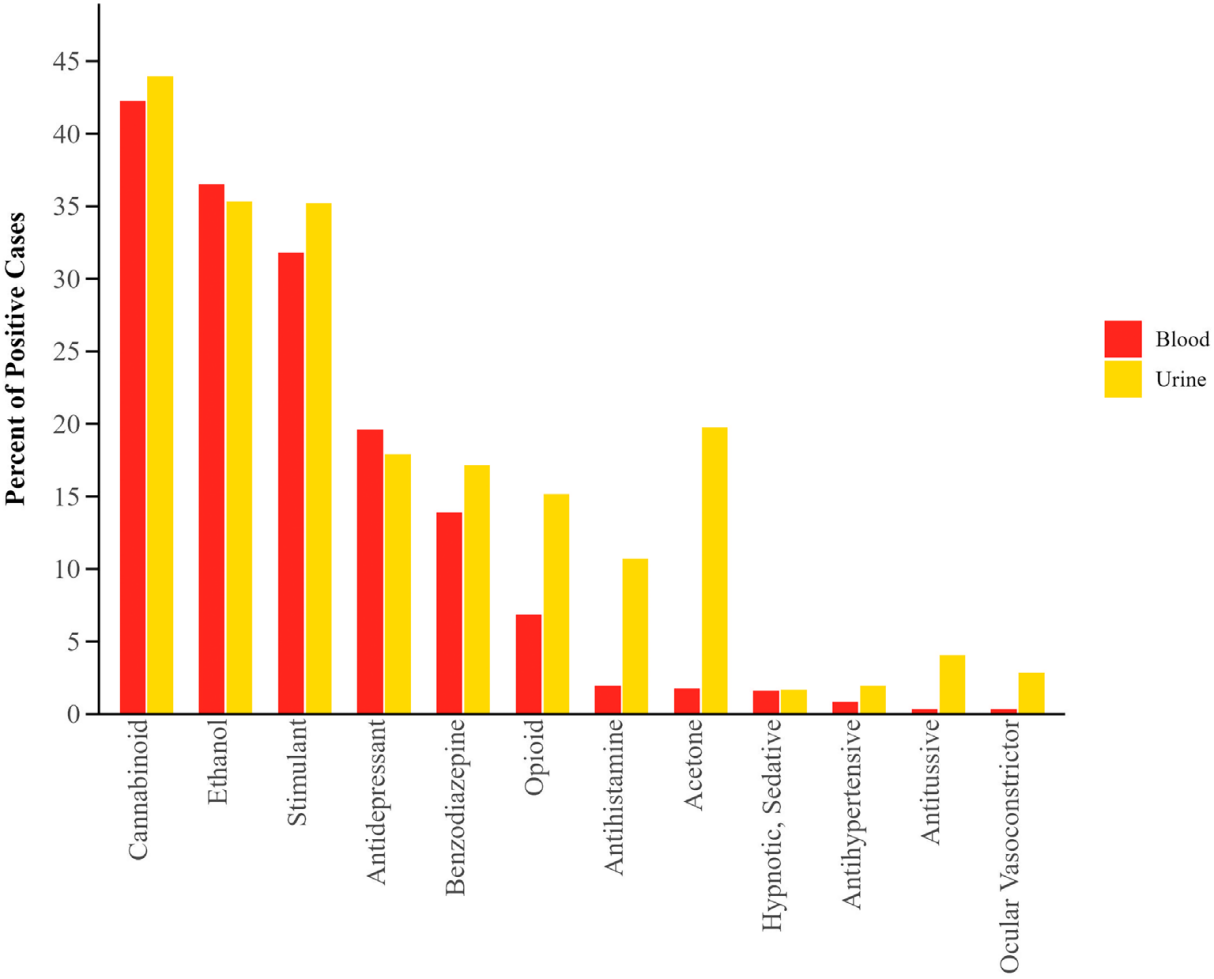
Frequency of drug class detection in positive blood (*N* = 2371) and urine (*N* = 4039) samples.

Ethanol remains a prevalent substance detected in DFC cases and is the second most prevalent substance by class noted in both blood and urine samples (Figure 3). Ethanol positivity and blood concentration ranges were evaluated by year, which can be viewed in Table S1.

Ethanol was found in 24% of blood samples (*n* = 565) and 28% of urine samples (*n* = 1427). Ethanol was noted as the only finding in blood in 14% of samples, and in urine, 12% of samples contained only ethanol, excluding drugs not known to have significant sedating or stimulatory effects. The average concentration of ethanol in blood submissions was 114 ± 78 mg/dL, with an approximate median concentration of 100 mg/dL and a range of 10–435 mg/dL. Average and median concentrations remained consistent each year.

Ethanol positive blood cases were evaluated to determine what other classes of substances were detected based on ethanol concentration (Table 6). Cases were counted in each group that was applicable to their positivity; for example, if a case contained ethanol, cannabinoids, and a benzodiazepine, it was counted in both the ethanol + cannabinoid group and the ethanol + benzodiazepine group. Co‐positivity was evaluated based on three ethanol concentration ranges: low (10–50 mg/dL), mid (51–79 mg/dL), and high (≥80 mg/dL). The highest number of cases was identified in the high ethanol concentration range overall, with 332 total cases, which is more than double either group. Ethanol was most prevalently found alone in 43%, 60%, and 48% of blood samples at low, mid, and high concentrations, respectively. The next most prevalent combination was ethanol in conjunction with cannabinoids, which was 18% or more of cases, with the highest percentage being the low concentration group (31%). Group concentration trends differ at the third categorization. Low ethanol concentrations and a stimulant were the third most prevalent (20%) while the third most prevalent with mid/high ethanol concentrations was an antidepressant with 12% and 15% of each group positivity for mid and high concentrations, respectively.

**TABLE 6:** Effect group combinations in ethanol positive blood cases based on ethanol concentration.

Ethanol group	Class combinations	*N*	% of ethanol group
Low (0.010–0.049) *N* = 137	Ethanol only	59	43
	Ethanol + Cannabinoid	43	31
	Ethanol + Stimulant	28	20
	Ethanol + Antidepressant	15	11
	Ethanol + Benzodiazepine	10	7.3
	Ethanol + Opioid	4	2.9
Mid (0.050–0.079) *N* = 95	Ethanol only	57	60
	Ethanol + Cannabinoid	17	18
	Ethanol + Antidepressant	11	12
	Ethanol + Stimulant	7	6.3
	Ethanol + Benzodiazepine	5	5.3
	Ethanol + Opioid	3	3.2
High (≥ 0.080) *N* = 332	Ethanol Only	160	48
	Ethanol + Cannabinoid	84	25
	Ethanol + Antidepressant	49	15
	Ethanol + Stimulant	39	12
	Ethanol + Benzodiazepine	31	9.3
	Ethanol + Opioid	11	3.3

### 3.5 Cannabinoids

Cannabinoids were the most prevalent substance detected across both matrices. Overall, c. 1/3 of blood and urine samples contained cannabinoids. Cases containing only cannabinoids accounted for 14% of blood cases (*n* = 325) and 12% of urine cases (*n* = 621).

### 3.6 Sedating substances

**FIGURE 4:**
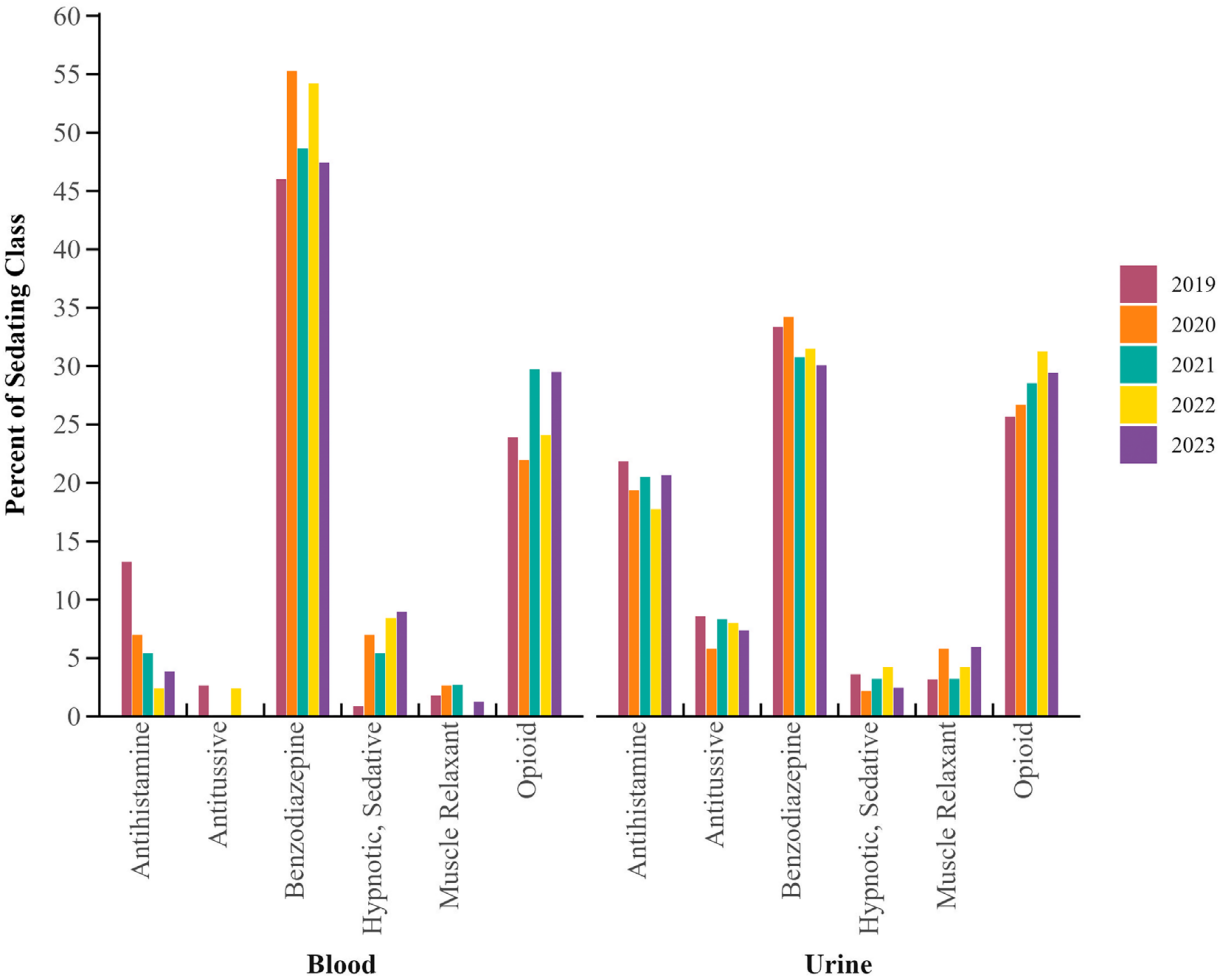
Percent positivity by drug class for all cases containing sedating substances in blood and urine by year.

Cases that contained one or more sedating substances accounted for 15% of blood specimens (*n* = 344). Only 3.8% (*N* = 91) of blood samples contained only a sedating compound. The most prevalent sedating substance, both class and analyte, was matrix specific (Figure 4).

One quarter of urine samples (*n* = 1512) contained a sedating drug. Other than alcohol, the top four sedating drugs detected in urine comprised four different drug classes: antidepressants, antihistamines, benzodiazepines, and opioids. Diphenhydramine was detected in 21% of samples containing a sedating substance. The next two most prevalent substances were fentanyl and lorazepam, accounting for 20% and 17% of group positivity, respectively. Analyte trends by year by matrix for benzodiazepines, opioids, and antihistamines can be found in Figures [Link], [Link], [Link].

### 3.7 CNS stimulants

**FIGURE 5:**
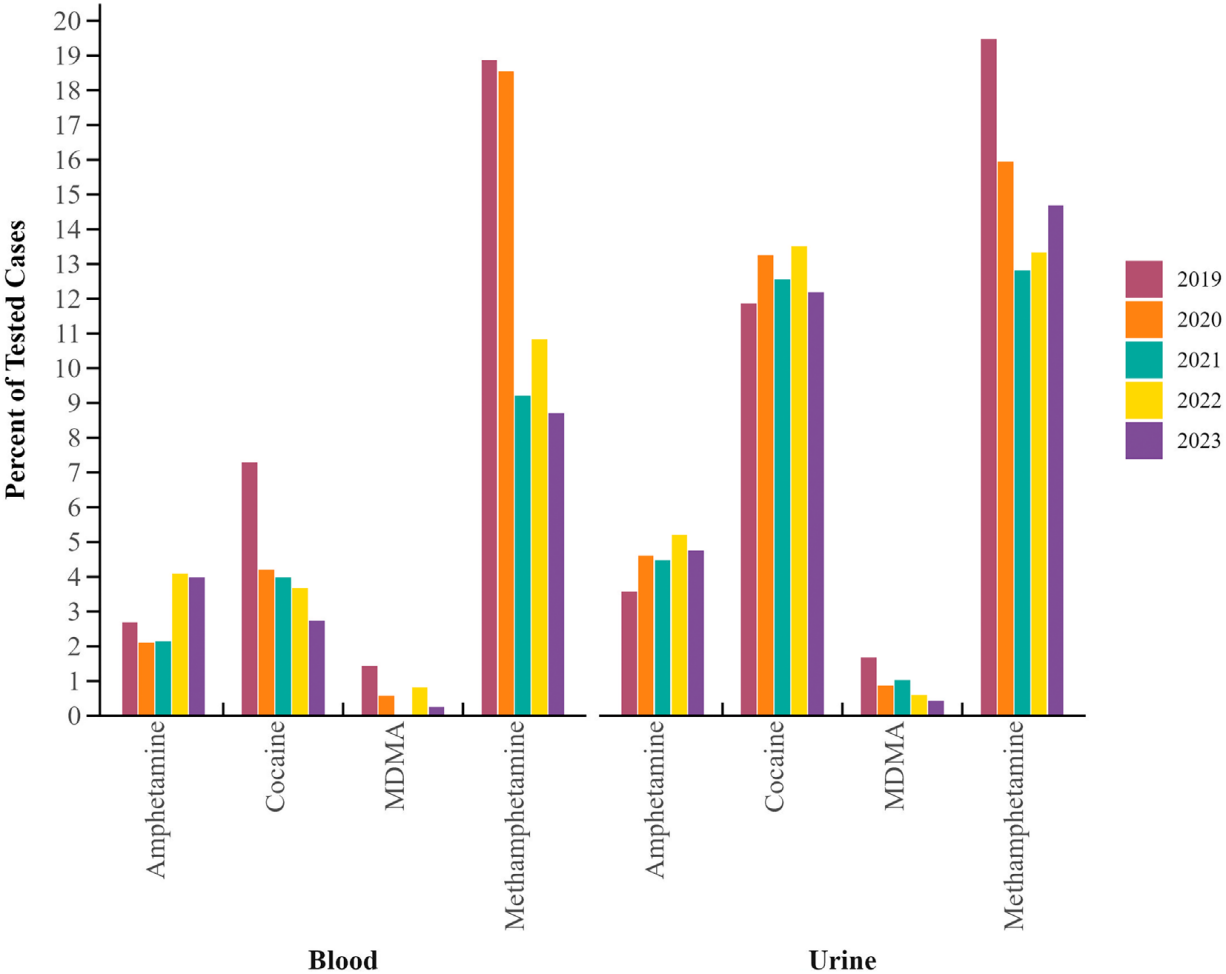
Percent positivity by stimulant analyte for all samples tested in blood and urine by year.

Stimulants were the least commonly detected effect group in blood samples (21%), high prevalence was still noted, which is depicted in Figure 5. In urine samples, stimulants were reported in 28% of cases, with 5.6% only containing a stimulant. Overall, 15% of urine samples contained methamphetamine, and cocaine positivity trailed slightly at 13%. Of note, between 2019 and 2023, a decrease in stimulant prevalence in both matrices for both cocaine and methamphetamine was noted. Methamphetamine decreased from c. 19% in 2019 to 12% in 2023 for both matrices. While blood cocaine prevalence was noted to decrease, no decrease in cocaine/metabolite positivity was observed in urine samples. MDMA/MDA urine positivity remained low at <1% across the 5‐year span; however, decreases were noted in 2019–2023 from 1.7% to <0.5%, respectively.

### 3.8 Effect group co‐positivity

The combinations of drugs were evaluated for positive samples. Four separate categories and their combinations: ethanol, cannabinoid, sedating substances, and stimulating substances were evaluated. For the purposes of evaluating drug combinations, analytes with no significant sedative or stimulant effect were excluded. Only 7.2% of positive blood samples and 8.3% of positive urine samples did not contain any analyte with one of these potential effects. Table 7 summarizes the number of positive cases that contained only analytes in the effect group combination, excluding cases that had drug effect group combinations as well as other substances. However, c. 1/2 of urine specimens contained only analytes in one group. Some combination of ethanol, cannabinoids, and/or sedating substances, with or without the presence of a stimulating substance, was found in 69% of urine samples (*N* = 2802).

**TABLE 7:** Combinations of effect groups reported in blood and urine samples.

Matrix	Effect group combination	*N*	% of positive cases
Blood	Cannabinoid only	325	21
	Ethanol only	323	21
	Stimulant only	186	12
	Cannabinoid + Ethanol	100	6.5
	Cannabinoid + Stimulant	99	6.4
	Sedating only	91	5.9
	Sedating + Stimulant	83	5.4
	Cannabinoid + Sedating + Stimulant	51	3.3
	Ethanol + Sedating	45	2.9
	Ethanol + Stimulant	42	2.7
	Cannabinoid + Sedating	35	2.3
	Cannabinoid + Ethanol + Sedating	24	1.6
	Cannabinoid + Ethanol + Stimulant	16	1.0
	Ethanol + Sedating + Stimulant	11	0.71
	Cannabinoid + Ethanol + Sedating + Stimulant	4	0.26
Urine	Cannabinoid only	621	15
	Ethanol only	608	15
	Sedating	367	9.1
	Sedating + Stimulant	286	7.1
	Stimulant only	280	6.9
	Cannabinoid + Sedating + Stimulant	256	6.3
	Cannabinoid + Stimulant	244	6.0
	Cannabinoid + Ethanol	225	5.7
	Cannabinoid + Sedating	222	5.5
	Ethanol + Sedating	165	4.1
	Ethanol + Stimulant	131	3.2
	Ethanol + Sedating + Stimulant	91	2.6
	Cannabinoid + Ethanol + Stimulant	82	2.0
	Cannabinoid + Ethanol + Sedating	73	1.8
	Cannabinoid + Ethanol + Sedating + Stimulant	52	1.3

Both a stimulatory substance and cannabinoids, combined with any other substance(s), were identified in 16% of urine positive cases. Six percent of urine samples contained solely the combination of a stimulatory substance and cannabinoids (Table 7). The minimum combination of cannabinoids and a sedating substance was found in 15% of cases (cannabinoid and sedating only—5.5%), and cannabinoids in combination with ethanol was found in 11% of cases (cannabinoid and ethanol only—5.6%). Cannabinoids, sedating drugs, and stimulants in the absence of ethanol were reported in 6.3% of cases, and 1.4% contained analytes from all four groups.

Ethanol in combination with only a sedating substance accounted for 4.1% of positive urine samples, while ethanol in combination with a sedating substance, as well as another drug, accounted for 24% of positive urine cases. By drug class, in conjunction with ethanol, benzodiazepines were identified to be the most prevalent drug class, closely followed by antihistamines.

Ethanol and one or more stimulating substances, in the absence of a sedating compound or Delta‐9 THC, were found in 2.7% of total blood samples (*n* = 42) and 3.2% of total urine samples (*n* = 131). In urine specimens, the most prevalent stimulants found with ethanol were cocaine/cocaine metabolites and amphetamines, which were reported in 55% and 60% of cases containing both ethanol and a stimulant.

### 3.9 Acetone

Acetone concentrations in blood were 5.1–18 mg/dL with a mean (±SD) and median of 9.1 ± 3.6 and 8.5 mg/dL, respectively. Five cases each contained acetone alone or acetone plus methamphetamine only. Acetone was detected in 16% of urine samples (*n* = 796). To provide a comparison group, acetone positivity was also evaluated in urine samples submitted for driving under the influence of drugs (*N* = 654) and medical legal death investigation cases (*N* = 2032); the positivity in both these groups was c. 6%. The average and median concentrations of acetone in urine were 26 ± 26 and 16 mg/dL, respectively, with a range of 5–180 mg/dL. Acetone in urine was most identified with cannabinoids, followed by being identified alone.

### 3.10 GHB

**FIGURE 6:**
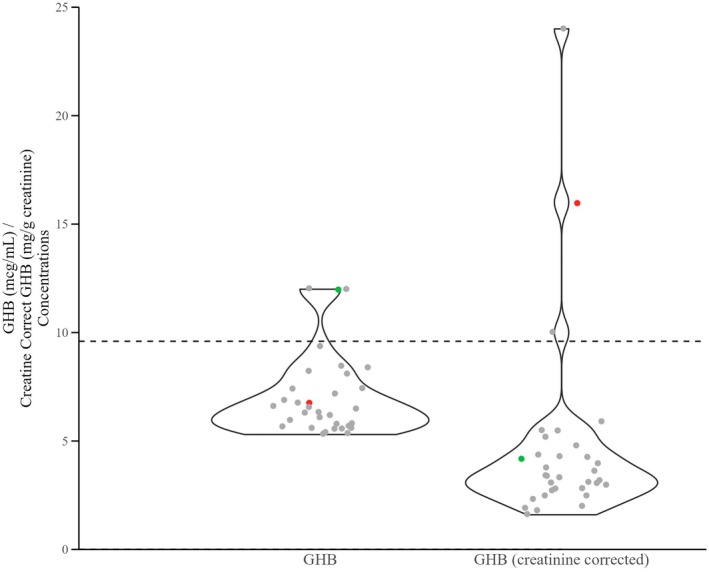
GHB raw and creatinine concentrations in urine. The dashed line marks the recommended GHB concentration to differentiate between endogenous and exogenous exposure of 9.6 mg/g creatinine. Green circles represent cases where the GHB concentration was greater than 10 mcg/mL but was less 9.6 mg/g creatinine after correction. Red circles represent a single case where the GHB concentration was less than 10 mcg/mL and was greater than 9.6 mg/g creatinine after correction. Twenty‐four cases with concentrations greater than 25 mcg/mL (25 mg/g creatinine) are not shown. Individual case paired data can be found in Table S2.

GHB was screened in all submitted samples. GHB was identified at a concentration ≥5 mcg/mL in <1% of blood samples (*n* = 18) and <1% of urine samples (*n* = 45). In urine samples, after results were creatinine corrected, only 19 of those cases had results >5.0 mg/g creatinine, and only 15 of those cases had results ≥10 mg/g creatinine (Table S2). GHB uncorrected concentration values versus creatinine corrected results were plotted to show very few cases exhibit urine GHB creatinine corrected results greater than 9.6 mg/g creatinine (Figure 6). Using the ASB required cutoff of 10 mcg/mL, very few cases were identified that could be associated with exogenous GHB administration.

## 4 DISCUSSION

The scope of analysis included analytes in ASB standard 121 and drugs that are part of the Society of Forensic Toxicologists Recommended Minimum Performance Limits for Common DFC Drugs and Metabolites in Urine Samples [9, 10]. The primary evaluation of results was regarding the ASB scope requirements and of those substances detected, most fell within the required scope. However, analytes outside of this scope which have sedating effects were also detected, including tetrahydrozoline, phencyclidine, methadone, and more. Additionally, changes were made to NMS Labs scope of analysis and reporting limits during the study period to achieve compliance with the ASB 121 reporting limits. Since the end of the study period, additional changes have been made to align with the ASB requirements, such as adding trazodone, mCPP, and norchlorcyclizine, which is a part of continuous quality improvement. Zolpidem 6‐carboxylic acid, which is also part of the ASB 121 requirement, has not yet been added to the DFC scope.

Current analytes that do not meet ASB scope requirements or required reporting limits, including zolpidem‐6 carboxylic acid and barbiturates, had minimal positivity. Zolpidem was analyzed for in urine cases, and only 12 cases were identified. In both blood and urine, the percent positivity of zolpidem did not exceed 0.5% on a yearly basis. Barbiturates were screened for at a threshold of 300 ng/mL, which is higher than the ASB required reporting limit of 100 ng/mL. Of the barbiturates evaluated for in both matrices, no positives were noted for pentobarbital; 21 positive cases were identified for phenobarbital, and four cases were identified for butalbital. Percent positivity never exceeded 0.75% for each year by matrix. Of the positive cases identified in urine, all exceeded concentrations of 100 ng/mL. Reported concentration ranges for butalbital were 250–2500 ng/mL, and phenobarbital were 1100–40,000 ng/mL, thus indicating a higher reporting limit for barbiturates may be sufficient for urine identification.

Novel psychoactive substances (NPS), including designer benzodiazepines and opioids, were not evaluated in this study because they are not in the current available scope. These substances should be evaluated as they have been reported in previous data sets, have impairing effects, and are proliferating the current drug markets. It is imperative that the recommendations be used as a starting guideline, and drug trends within the region of incident be routinely evaluated to determine whether they should be added into each laboratory's scope of testing.

### 4.1 General case evaluation

Preanalytical factors, including sample collection protocols, sample matrix collected, the time interval between the incident and sample collection and the scope and cutoff for testing are also critical aspects impacting the weight that should be given to toxicological evidence in these investigations. Sample collection postincident should occur as quickly as possible after a person becomes aware or suspects they may have been a victim of a DFC. A delay in incident reporting, during which drug metabolism or elimination can occur, increases the likelihood of a negative toxicology report. An Australian study reported that the median delay time for reporting an alleged DFC incident to the time of sample collection was 20 h, however, reporting windows varied greatly [4]. Upon receiving a report of a suspected DFC, a knowledgeable person such as a specially trained police officer, an emergency room doctor, a SANE, a forensic or clinical toxicologist, or a public health resource guide should be consulted to maximize the likelihood of a meaningful interpretive test. Any postevent drug use or medical drug administration should be recorded and documented. Sample matrix selection should be tailored to each case based on the time interval between incident and collection and information about the pharmacokinetics of drugs of interest if known. Blood collection is recommended with small time intervals (0–24 h) between the event and the sample collection, whereas urine collection is recommended when a longer interval has passed (12–120 h). If there is an overlap, or if the time of incident is unknown, both specimens should be collected [4]. If the event occurred days or weeks previously, it has been shown that hair collection may be a viable sample matrix [13, 14], but this requires careful interpretation by a person familiar with the pharmacokinetics of drug deposition in hair. Within this study, very few cases provided collection interval information, thus limiting the trend interpretation conclusions able to be drawn.

Previously reported positivity studies of DFC cases list ethanol as the most prevalent substance detected, which is consistent with popular media reports [15, 16, 17, 18]. However, these reports are frequently not current or comprehensive. These reports also conflict with this study's findings, where cannabinoids were the most prevalent finding, followed by ethanol. The United States Department of Justice Drug Enforcement Agency (DEA) authored a document in 2017 on drug facilitated sexual assault (DFSA) and common substances to be aware of [19]. The substances of interest used in DFSA, as reported by the DEA, include flunitrazepam (Rohypnol®), GHB, and ketamine [16, 17, 18, 19], which, while capable of impairing the ability to consent, are very infrequently used for this purpose compared to other more common drugs, based on current data. Very few incidents of positive cases have been noted for either ketamine or GHB in recent years, especially in the United States, although they continue to attract disproportionate media attention [15, 16, 18, 20, 21].

In this study, a decrease in cases for both blood and urine samples in 2021 was noted, which coincides with a lower percent positivity of substances detected. COVID‐19 resulted in closures across the United States around March 2020 for a mixture of bars, restaurants, and other gathering areas of individuals. It is generally noted that in natural disaster scenarios, sexual assault and sexual violence reporting increases, which was also observed during this period via hotline calls [22]. Even with the increased calls, a decrease in sexual assault reporting to law enforcement was noted during the COVID‐19 pandemic [23]. It is possible that the lower 2021 case submissions were a residual effect of the implications of the COVID‐19 pandemic and decreased reporting of DFC cases.

### 4.2 Demographics

Within the United States, an individual becomes a victim of sexual violence approximately every minute [3]. Those who are at highest risk of rape and sexual assault are individuals 18–34 years of age, followed by those who are 35–64 years old [3]. Adults being the most prevalently identified age group has been supported in published literature as well [5, 24, 25], which is consistent with this study. The likelihood of rape and/or sexual assault decreases twofold for adults in comparison to younger individuals. Seniors 65 years or older are least likely to become victims of sexual assault/rape [3]. In 2016, Child Protective Services (CPS) agencies found that over 57,000 children were victims of sexual abuse [26]. Of all victims under the age of 18, children aged 12–17 comprised two‐thirds of reported sexual assaults or rapes, and females aged 16–19 are on average four times more likely to be a victim of rape (completed or attempted), or sexual assault when comparing to general populations [26]. Over 90% of study patients were female, which is consistent with the statistics reported in sexual assault and rape cases reported by the CDC [1].

The most commonly detected drug in children was fentanyl, followed by methamphetamine and midazolam. The presence of these drugs in children does not necessarily indicate drug administration prior to the assault. Methamphetamine can be detected in the hair or urine of children who live in homes where these drugs were manufactured and/or used via environmental exposure or accidental ingestion. Wright et al. evaluated 25 case studies involving residents in homes previously used for methamphetamine manufacture or occupied by methamphetamine users. Hair samples were tested from 36 participants, 20 of whom were under 12 years of age. Eleven of the 20 participants under 12 years of age had methamphetamine‐positive hair samples at a cutoff of 5 pg/mg. The authors also noted that higher levels of exposure were noted in younger children, which is consistent with exposure via playing on the floor and putting contaminated objects and hands in the mouth [27]. Castaneto et al. evaluated 91 children ages 1 month to 18 years with household methamphetamine exposure. Hair, urine, and oral fluid were tested [28]. All three children under the age of 12 months tested positive for methamphetamine in at least one specimen; 74% of participants ages 1–18 years had a positive sample. Over 75% of hair samples were positive for methamphetamine, and c. 21% of urine samples contained the drug at concentrations of 25.1–107.8 ng/mL. Based on these data, it is not possible to rule out environmental exposure based solely on a toxicology report. Pediatric fentanyl deaths increased 30‐fold between 2013 and 2021 [29] and the number of illicit fentanyl exposures reported to the national poison data system in children under the age of 6 increased from 5 in 2013 to 539 in 2023 [30]. Fentanyl and midazolam may be used in the emergency department or outpatient clinics for sedation and analgesia during invasive medical procedures [31]. Hogan et al. examined the use of midazolam for conscious sedation in children undergoing sexual assault examinations and found it to be safe when sedation is needed [32].

### 4.3 Blood and urine combinations

A total of 1361 cases in this data set were analyzed, including blood and urine paired samples. In c34% of cases, analyte identification was consistent between paired blood and urine samples. For urine samples specifically, c. 63% had at least one analyte detected in urine that was not identified in blood from the same case. Overall, 22% of cases had urine positive results with negative blood results. Many of the substances with discrepancies between urine and blood samples, such as ethanol and cocaine/metabolites, are noted to have extended windows of detection and also allow for the analysis of drug metabolites, which could explain select matrix‐specific discrepancies. Cannabinoid positivity in blood and urine were very similar (28% and 35%, respectively), even though the detection window for the metabolite in urine following a single use would be longer than that in blood [33, 34, 35]. In chronic users, positive blood cannabinoid cases have been identified 28 days postuse [33, 34], which could also explain positivity. The screening reporting limit in urine was higher than in blood as well. Without case history and last use provided in all these cases, and information regarding regular use, further conclusions could not be made. With these data combined, it is imperative that at minimum, urine be collected in DFC cases due to analyte identification discrepancies between matrices. Collecting blood samples may be beneficial in addition to urine collection, especially if a shorter detection window between incident and collection is noted. Regardless of the situation, it is beneficial to collect urine due to detection window and analyte choices, thus allowing an increased likelihood of substance detection and a more accurate toxicological representation of a case.

### 4.4 Ethanol

Ethanol is a primary substance of concern in DFC cases. Ethanol was the second most prevalent substance identified in blood and urine cases. Ethanol was identified in c. 35% of positive blood and urine samples, which is consistent with previously reported literature [8, 36]. The average and median concentrations of ethanol in blood samples observed minimal change over all 5 years of the study period, even when the number of cases and the number of positive cases identified had minor fluctuations.

Ethanol has CNS depressant effects that can impair judgment and cause disinhibition in individuals consuming the substance [5, 37]. Effects may be more pronounced at higher blood alcohol concentrations and can include memory impairment, muscular incoordination, and coma [5, 37]. These effects make ethanol a common substance of choice in proactive DFSA, as well as opportunistic DFSA, as consent may not be able to be provided. The effects of ethanol in conjunction with another substance can also be more pronounced [5]. When examining ethanol concentration ranges in blood samples and co‐positivity of other substances, ethanol concentration did not seem to affect co‐positivity of other substances for the most prevalent combinations. Most commonly, ethanol was found alone, followed by in conjunction with cannabis. Cannabinoids have a wide range of effects including depressant effects such as sedation, loss of coordination, disorientation, and drowsiness, all of which can cause impairment [38]. These effects can be at least additive when combined with ethanol [38, 39, 40, 41, 42]. Ethanol was also frequently identified with benzodiazepines, specifically alprazolam, lorazepam, and clonazepam/7‐aminoclonazepam, which is consistent with the most commonly prescribed benzodiazepines in the United States [43]. The third most prevalent combination included ethanol, and either an antidepressant or a stimulant, specifically methamphetamine. The top three antidepressants detected were classified as nonsedating antidepressants in the selective serotonin reuptake inhibitor (SSRI) category: citalopram/escitalopram, fluoxetine/norfluoxetine, and sertraline/desmethylsertraline. While these substances were detected frequently, they have little bearing on DFC case interpretation due to their lack of sedating effects.

### 4.5 Cannabinoids

Cannabinoids were the most prevalent drug class detected in both blood and urine samples at 42% and 44%, respectively. Delta‐9‐THCCOOH is known to have varying detection windows, which can be prolonged by chronic delta‐9‐THC administration; that, in conjunction with increased rates of recreational use, could explain this finding. While cannabinoids can be sedating, identifying when cannabis was last used by an individual, based on blood or urine concentrations of delta‐9‐THC or its metabolites, is not possible.

Per the CDC as of 2021, cannabis remains the most prevalent federally illegal substance used in the United States [44]. More than 52 million people have used cannabis at least once [44]. Delta‐9 THC can remain in the body and be detected in the blood for many hours post‐use, and even longer for chronic users in blood and plasma samples [45]. Urine samples have noted detection windows exceeding 2 weeks for chronic cannabis users, even after a period of abstinence [35]. It is imperative that a positive cannabis finding is evaluated within the case‐specific circumstances and an individual's specific drug use history, as a positive finding would not necessarily be related to a DFC incident due to the extended detection windows in each matrix for chronic users.

### 4.6 Sedating substances—Benzodiazepines

Legacy benzodiazepines decreased in percent positivity across the study period in both matrices, specifically for alprazolam and clonazepam. Also, the United States has noted some of the lowest percent positivity in legacy benzodiazepine positivity as well as multiple different benzodiazepines being detected [46]. Diazepam trends remained consistent, with a low percent positivity overall. Lorazepam had an increase in positivity in blood samples by c. 2%; however, there was a slight decrease in positivity in urine samples. While an overall decrease was noted for this drug class, it is critical that they continue to be tested for due to their overall positivity rates and the significance of their potential effects. Benzodiazepines can have relevant effects even at low doses in subjects, can be administered surreptitiously in spiked drinks, and quickly exhibit their effects [47]. Some benzodiazepines also have short half‐lives which may decrease their detectability in both blood and urine matrices due to detection window concerns and delays in reporting [47]. Effects of benzodiazepines on an individual include sedation and relaxation, as well as drowsiness, slurred speech, confusion, motor incoordination, retrograde amnesia, and disinhibition, which make them a drug of choice for DFCs [47]. Often, case history is not provided during toxicology analysis, and midazolam or another benzodiazepine may be given in a hospital setting postincident for the treatment of the victim [48]. It is important to note that this could affect the percent positivity of analytes detected. Designer benzodiazepines have become prevalent in other monitored populations, such as individuals driving under the influence of drugs (DUID), with a variety of novel illicit benzodiazepines detected [49]. Designer benzodiazepine presence has also been reported in DFSA cases, including the United States, Canada, and selected locations in Europe [47]. Designer benzodiazepines were not in the scope of analysis for this study, which likely causes underreporting.

### 4.7 Sedating substances—Opioids

Overall opioid positivity showed increases that were not matrix specific. Blood positivity noted variability between years, whereas urine matrices noted a slow increase over time, with positivity being c. 5% and 12%, respectively. Urine opioid percent positivity is comparable to previous data, whereas the blood positivity trended lower [8, 50]. The most prevalent opioid noted in blood was fentanyl, which exceeded the positivity rate for all other opioids. Fentanyl was also the most prevalent substance identified in urine case submissions for children. The positivity rate of fentanyl, however, never exceeded 3% in blood or 7% in urine during the study, despite much higher positivity rates in medico‐legal death investigations and DUID cases. Previous literature notes codeine, morphine, and hydromorphone to be some of the most common opioids of interest; however, these compounds were not as prevalent in comparison to fentanyl [8, 46, 50]. Other opioids of interest following fentanyl include dextro/levo methorphan and hydromorphone in urine as well as methadone and oxycodone in blood samples.

Opioid interpretation should be evaluated with caution as opioids can be prescribed licitly for pain management. Pain, both acute and chronic, is commonly noted in individuals who have experienced rape, and individuals can be prescribed opioids as a part of their treatment [51]. Information about a person's prescription history, as well as not knowing whether toxicology samples were taken pre or post administration of pain management care, is necessary for the appropriate interpretation of the potential role of opioids in a crime.

### 4.8 Sedating substances—Other

Other sedating substances, including various antidepressants, antihistamines, and antitussives, were found within the data set and were of interest in the findings. Antidepressants were the fourth most prevalent drug class noted in blood samples and fifth in urine samples; however, the top three antidepressants noted were all nonsedating SSRIs, which may not be impactful to case interpretation. It is possible that SSRI findings could be present due to daily prescribed dosing regimens of the victim of an incident.

Dextromethorphan/levomethorphan was detected in c. 3% of cases. While the analysis performed does not differentiate between enantiomers, levomethorphan, an opioid receptor agonist, is rarely prescribed, and both enantiomers have potential impairing effects. Urine samples also remained consistent in percent positivity across the 5‐year analysis period. Percent positivity remained low within blood samples with minimal detections. Some studies did not test for methorphan and its metabolite, whereas others grouped it with opioids [36, 50, 52]. A previous study examined percent positivity of methorphan and its metabolite and saw similar percent positivity as noted in this study [8].

Antihistamines in blood samples had low positivity, which did not exceed 3%. In urine, antihistamines were more frequently detected, with diphenhydramine positivity ranging from c. 4.5%–8%, depending on the year. Excluding 2019, which had a low positivity rate for chlorpheniramine, diphenhydramine was the only antihistamine detected in blood during the study period. Doxylamine and chlorpheniramine were also noted in urine cases; however, with lower positivity not exceeding 2.5% and 1.3% positivity, respectively. Diphenhydramine has been noted in a similar percent positivity in DFC cases across multiple studies, ranging from 2% to 6.4% [8, 50]. Antihistamines, specifically first‐generation antihistamines, exhibit sedation and performance impairment [53], thus making them ideal substances for DFC. However, many antihistamines are also available as over the counter (OTC) medications, including formulations as sleep aids, increasing their availability. Common antihistamines may also be taken therapeutically to treat allergies or used as a sleep agent or as a remedy for motion sickness, which may also increase prevalence [53].

### 4.9 Stimulating substances

Very few stimulants were noted to have positive findings within DFC cases. The most prevalent stimulant noted was methamphetamine, closely followed by cocaine. Prevalence trends were not matrix dependent. Cocaine prevalence remained relatively consistent in urine, while minimally lower in blood, than rates in previously reported literature [8, 36, 52]. Methamphetamine demonstrated a decrease in positivity from previously reported trends [8], while MDMA (aka ecstasy) maintained minimal positivity, which is consistent, if not lower, than previously reported literature [8, 36, 52, 54]. While methamphetamine positivity has declined, it is still a prevalent finding in DFC cases and consistent with previous literature where it was one of the most prevalent substances identified within the stimulants category and in combination with other drugs [8, 54]. Methamphetamine may have decreased in prevalence overall due to other substances that have become prevalent over the years, mainly opioids including fentanyl. Other substances, such as NPS, have become popular in recent years in illicit drug markets and usually have impairing effects associated with them. As they were not screened for within this sample population, we have no data on their prevalence in DFC. Other populations, including DUID and postmortem cases, have demonstrated increases in NPS compounds [49, 55, 56, 57].

### 4.10 Flunitrazepam and GHB

Flunitrazepam and GHB are two substances commonly associated with DFC by the public, the media, and by healthcare professionals; however, flunitrazepam and its metabolite were detected in only one case of total submissions, calling into question its continued use as a DFC agent—especially since it has never been approved for medical use in the United States [8, 36]. Flunitrazepam is not only frequently discussed in media reports but also cited as one of the common substances of concern by the DEA as a DFC agent due to its short duration of action, short detection window, and impairing and amnestic effects [19]. Despite short detection windows (which may limit its detection due to a delay in reporting an incident), even ASB Standard 121 does not recommend including flunitrazepam in its general scope of analysis due to its low prevalence [9].

GHB is an endogenous substance commonly encountered in living individuals. Threshold guidelines set forth by ASB exist to attempt to separate endogenously produced GHB from exogenous administration. Various studies have suggested different thresholds for distinguishing between endogenous versus exogenous GHB, ranging from 5 to 20 mcg/mL [58, 59, 60, A. Eklund and M. Eriksson, Personal Communication, TIAFT, Helsinki (2000).]. Those studies also point out that analytical methods account for total GHB and GBL due to pH dependent equilibrium/interconversion [58]. The method used for the analyses reported does meet this requirement. Creatinine correction has been recommended in urine DFC cases to account for hydration or dehydration, which can be accounted for by normalizing the reported GHB value to creatinine [61]. Crookes et al. [60] recommend a GHB cutoff of 5 mcg/mL and a creatinine corrected GHB cutoff of 1000 mg/mmol (9.6 mg/g creatinine). While multiple threshold recommendations to distinguish between endogenous vs. exogenous GHB do exist, it appears that 10 mcg/mL is the established accepted threshold [10, 58, 59, 61]. There are additional factors that can cause increases in GHB levels that must be accounted for prior to interpreting the final GHB concentration. Warmer storage conditions (25°C) as well as freeze–thaw cycles, have caused increased GHB concentrations [9, 58, 59, 61]. Another situation that can cause increases in urinary GHB levels is GHB aciduria, which is an uncommon genetic disorder characterized by a succinic semialdehyde dehydrogenase deficiency that can increase endogenous urinary GHB concentrations to levels greater than 10 mcg/mL [58, 62].

Adopting a threshold recommendation of 10 mcg/mL in both blood and urine, <1% of GHB detections in either blood or urine in this study population suggest exogenous administration. Two cases in urine, after creatinine correction, changed interpretation for exogenous versus endogenous as the final reported result either increased above 10 mg/g creatinine (case 14) or decreased to exactly 10 mg/g creatinine (case 15) after correction, demonstrating the value of creatinine correction in urinary GHB analysis to account for urinary dilution due to dehydration or over hydration. Three cases contained between 10 and 25 mg/g creatinine, and two cases contained between 26 and 50 mg/g creatinine in urine. While these five cases met or exceeded the recommended GHB concentration threshold, no information was available regarding the disease states of the subject or the pH or storage conditions prior to reporting. In cases with GHB concentrations that make it challenging to differentiate between exogenous administration vs. endogenous levels, urine re‐collection at a later date has been suggested to establish an individual's baseline GHB concentration to provide additional context for interpretation [62]. If re‐collecting, additional results must be interpreted with caution as individuals experience baseline urinary GHB concentration fluctuations daily and at different time points [61]. However, if an individual's test results show a urinary GHB concentration between 10 and 25 mg/g creatinine, there may be some benefit in testing a later sample at the same collection time for additional context. Applying the recommended cutoff of 5 mcg/mL GHB and 9.6 mg/g creatinine proposed by Crooke et al. [60] results in the interpretation for all but two positive cases changing from exogenous to endogenous.

Two limitations to interpreting GHB concentrations in both matrices in this case series are that the collection interval and storage conditions prior to sample submission were not available; however, GHB was tested for in all cases. Urine samples were stored in frozen temperature conditions to help minimize preanalytical factors [59]. A nondetected GHB result should also be interpreted with caution, especially without understanding the full picture, as a negative result does not necessarily mean the substance was not present at the time of the incident, only that it was not detected when analyzed for.

### 4.11 Drug interactions

In this study, c. 3 quarters of all case submissions tested positive for more than one potentially impairing substance, which could compromise an individual's ability to provide consent. In both blood and urine samples, cannabinoids with ethanol were found to be the most prevalent combination, followed by cannabinoids with methamphetamine. The third most prevalent combination of substances in blood samples was ethanol with methamphetamine, whereas in urine samples, the third most prevalent was cannabinoids with cocaine/cocaine metabolites.

In terms of effect profiles, cases frequently contained a cannabinoid (which can have a sedating effect) alongside a CNS depressant or a CNS stimulant. Typical CNS depressant effects include relaxation, drowsiness, dizziness, and cognitive effects including impaired memory function and attention. With stimulants, there are two effect profiles: the initial stimulatory phase and the secondary withdrawal phase. The initial stimulatory phase will typically present with increased alertness, euphoria, excitation, agitation, and rapid speech. The secondary withdrawal phase mimics effects associated with CNS depressants, including lethargy, sedation, and drowsiness. Without observations of the individual, there is no way to determine from a drug concentration which phase an individual was in at the time of sample collection. With the combination of multiple substances, there can be either additive or synergistic effects, dependent upon the drug–drug interactions, thus potentially increasing the effects an individual may experience while under the influence of a substance [62].

### 4.12 Acetone

Acetone was analyzed in both blood and urine samples. In blood samples, a positivity rate of 1.1% was found; in urine c. 16% of samples were positive, which is c. 2.6 times higher than the prevalence in DUID cases. Acetone prevalence is consistent with previously reported literature where acetone positivity was examined in DFSA‐specific cases [63]. Arndt et al. reported that 11% of DFC cases contained acetone in at least one matrix. Acetone presence may be attributed to either consumption or endogenous production, including diabetes, dietary composition, alcoholism, and stress response. DFSA cases are understood to cause added stress to the victim based on the nature of the crime; thus, acetone findings could support an elevated stress response [63]. Limited case‐specific information on the victim and crime prohibits ruling out disease states or consumption as an alternate reasoning for acetone detection and prevalence.

## 5 CONCLUSIONS

From 2019 to 2023, over 6000 DFC cases were analyzed to determine drug trends, the largest data set to date. For each case, urine, blood, or both were tested, with the analysis of results focused on urine since that is the recommended matrix for DFC casework; however, a comparison between blood and urine results was included to determine if the results were consistent with the recommendation.

Among matched blood and urine specimens, analyte positivity appeared to be matrix dependent, with additional analytes reported in urine that were not detected in blood. Urine allows for longer detection windows of parent drug as well as the ability to analyze for drug metabolites, increasing the likelihood of detecting a potential substance, even from a single use. This supports urine as the preferred matrix in DFC investigations; however, it is important to recognize the interpretive value of blood results over those of urine results with respect to impairment.

In contrast to previous literature, cannabinoids were identified to be the most prevalent drug class and had overtaken ethanol in positivity. This is no doubt reflective of general increases in rates of cannabis use since its decriminalization or legalization in many US states. Sedating substances, including benzodiazepines, are known analytes of interest in DFC cases due to the sedating and impairing effects they can impart on a victim and compromise someone's ability to provide consent. In addition, many cases contained several different sedating substances, which emphasizes the importance of considering drug–drug interactions in these investigations. Stimulatory substances were also identified less frequently than other classes, but they maintained high positivity, with top findings including methamphetamine and cocaine/metabolites. Ethanol, flunitrazepam, GHB, ketamine, and MDMA are all substances commonly associated with DFC cases. Aside from ethanol, the above‐mentioned drugs were rarely detected within analyzed case submissions across the 5‐year analysis period.

Case history, including collection time interval and prescription records if available, is essential in interpreting the results of DFC testing, to help determine whether analyte positivity was a result of a DFC, as a result of treatment post‐incident, or present and unrelated to the crime. Prescription and OTC medications taken as directed by a medical professional, as well as substances present due to an individual's total body burden from chronic use, may be observed. A perpetrator may be aware that an individual is taking a prescribed medication or another impairing substance willingly, and taking advantage of that situation based on the effects that substance has on the victim. They may also dose a victim unknowingly and unwillingly with a prescription medication. Finally, the lack of a substance being detected does not necessarily mean that one was not initially present at the time of incident due to reporting delays or said substance's short detection window. It is imperative when interpreting toxicology findings in DFC cases that they be evaluated on a case‐by‐case basis and put into the perspective of the case, which includes scene investigation information; toxicology remains one facet in a DFC investigation.

The current scope of testing is based on requirements from ASB standard 121 as well as SOFT recommendations, in addition to including other commonly encountered substances. However, the drug markets are constantly changing, and new substances are increasing in popularity at alarming rates. Current literature notes an increase in designer benzodiazepine prevalence in both DUID and DFC communities, and this may be a group of substances worth adding based on the impairing effects they may impart on an individual. Future work includes analyzing authentic DFC case samples for the presence of designer benzodiazepines and other impairing designer compounds to further recommend whether these should become part of the routine DFC scope of testing based on positivity, as well as working to incorporate them into current panels. It is imperative that scopes continue to be re‐evaluated and updated in a timely manner to account for the ever‐changing drug markets and substances available to illicit impairing effects that could compromise an individual's mental and physical faculties.

## CONFLICT OF INTEREST STATEMENT

The authors have no conflicts of interest to declare.

## DISCLAIMER

All authors are paid employees of NMS Labs.

## Supporting information


**Table S1.** Number positive (N), mean ± standard deviation, median and concentration ranges (mg/dL) of ethanol in blood specimens.
**Table S2.** Gamma‐hydroxybutyric acid (GHB) concentrations in urine positive samples with and without creatinine correction.

**Figure S1.** Percent positivity by benzodiazepine analyte of for all samples tested in blood and urine by year.

**Figure S2.** Percent positivity by opioid analyte of for all samples tested in blood and urine by year.

**Figure S3.** Percent positivity by antihistamine analyte of for all samples tested in blood and urine by year.

## Data Availability

The data that support the findings of this study are available from the corresponding author upon reasonable request.
