# Supplementary material for: Drug‐facilitated crime: A review of findings between 2019 and 2023
Source: J Forensic Sci. 2025 Aug 12;70(6):2442–64. doi: 10.1111/1556-4029.70151 (PMC12584120; doi:10.1111/1556-4029.70151)
Supplement: Supplementary file 1 — Table S1. Number positive (N), mean ± standard deviation, median and concentration ranges (mg/dL) of ethanol in blood specimens. Table S2. Gamma‐hydroxybutyric acid (GHB) concentrations in urine positive samples with and without creatinine correction. [file JFO-70-2442-s003.docx]

TABLE S1 Number positive (N), mean ± standard deviation, median and concentration ranges (mg/dL) of ethanol in blood specimens.

| Year | N | Mean | Median | Range |
| --- | --- | --- | --- | --- |
| 2019 | 155 | 110**±**72 | 101 | 10 - 344 |
| 2020 | 138 | 113**±**79 | 91 | 10 - 435 |
| 2021 | 83 | 119**±**84 | 98 | 10 - 362 |
| 2022 | 125 | 107 **±**76 | 86 | 10 - 421 |
| 2023 | 119 | 123**±**82 | 113 | 10 - 402 |

TABLE S2 Gammahydroxybutyric acid (GHB) concentrations in urine positive samples with and without creatine correction.

| **Case Number** | **GHB Concentration**  **(mcg/mL)** | **Creatinine Corrected Concentration**  **(mg/ g Creatinine)** |
| --- | --- | --- |
| Case 1 | 2200 | 4900 |
| Case 2 | 2000 | 3000 |
| Case 3 | 1300 | 1200 |
| Case 4 | 390 | 1100 |
| Case 5 | 800 | 840 |
| Case 6 | 780 | 720 |
| Case 7 | 350 | 690 |
| Case 8 | 470 | 250 |
| Case 9 | 150 | 180 |
| Case 10 | 240 | 120 |
| Case 11 | 83 | 47 |
| Case 12 | 81 | 37 |
| Case 13 | 12 | 24 |
| Case 14 | 6.8 | 16 |
| Case 15 | 12 | 10 |
| Case 16 | 5.6 | 5.9 |
| Case 17 | 5.8 | 5.5 |
| Case 18 | 8.1 | 5.5 |
| Case 19 | 7.4 | 5.2 |
| Case 20 | 5.8 | <5.0 |
| Case 21 | 8.5 | <5.0 |
| Case 22 | 5.3 | <5.0 |
| Case 23 | 6.6 | <5.0 |
| Case 24 | 12 | <5.0 |
| Case 25 | 6.8 | <5.0 |
| Case 26 | 5.6 | <5.0 |
| Case 27 | 6.6 | <5.0 |
| Case 28 | 6.5 | <5.0 |
| Case 29 | 6.2 | <5.0 |
| Case 30 | 6 | <5.0 |
| Case 31 | 5.7 | <5.0 |
| Case 32 | 6.9 | <5.0 |
| Case 33 | 8.4 | <5.0 |
| Case 34 | 9.4 | <5.0 |
| Case 35 | 5.6 | <5.0 |
| Case 36 | 7.4 | <5.0 |
| Case 37 | 7.2 | <5.0 |
| Case 38 | 6.3 | <5.0 |
| Case 39 | 5.4 | <5.0 |
| Case 40 | 8.2 | <5.0 |
| Case 41 | 5.6 | <5.0 |
| Case 42 | 6.3 | <5.0 |
| Case 43 | 5.4 | <5.0 |
| Case 44 | 6.1 | <5.0 |
| Case 45 | 5.7 | <5.0 |
