# Supplementary figures and images for: Drug‐facilitated crime: A review of findings between 2019 and 2023
Source: J Forensic Sci. 2025 Aug 12;70(6):2442–64. doi: 10.1111/1556-4029.70151 (PMC12584120; doi:10.1111/1556-4029.70151)

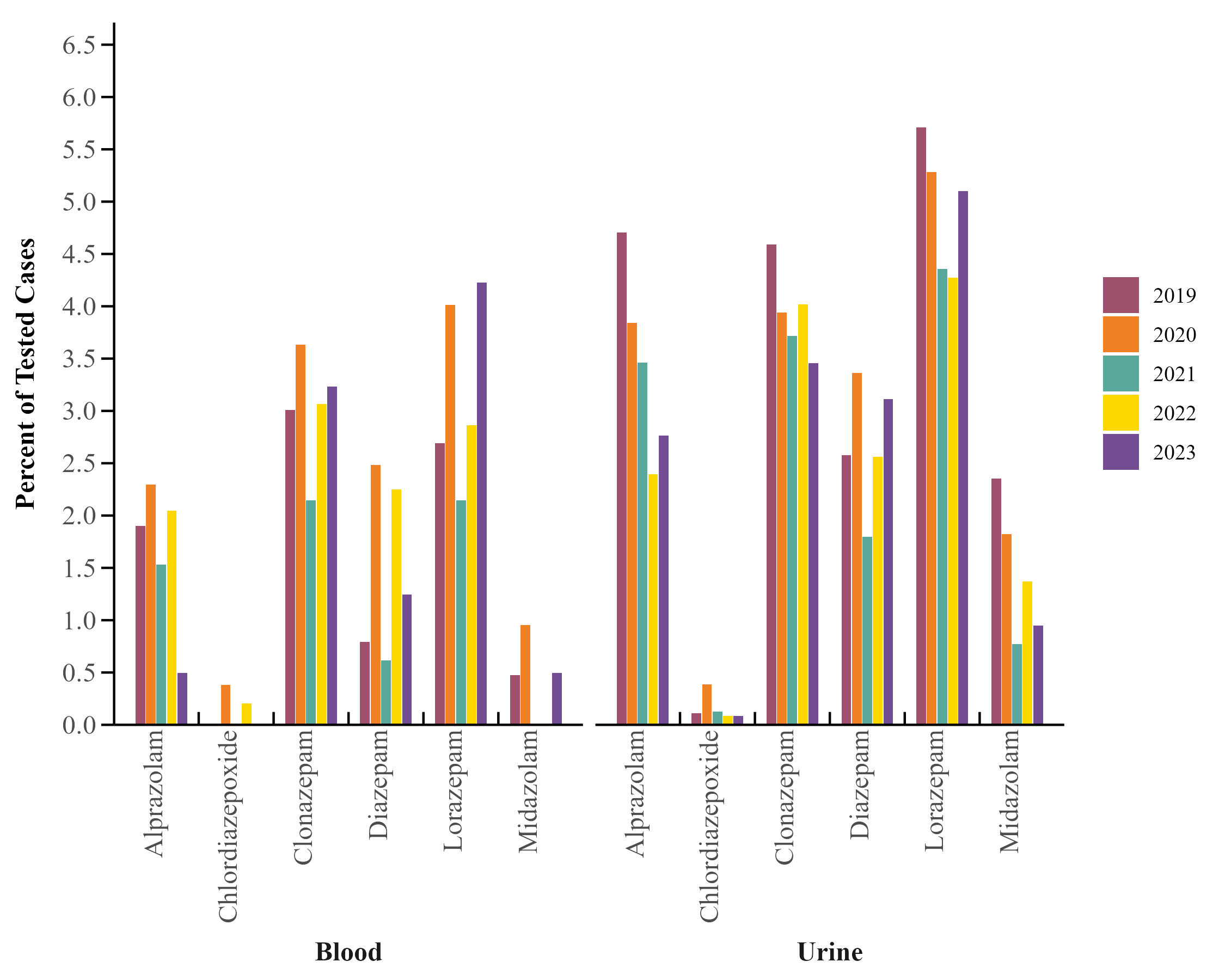

Supplement: Supplementary file 2 — Figure S1. Percent positivity by benzodiazepine analyte of for all samples tested in blood and urine by year. [file JFO-70-2442-s002.jpeg]

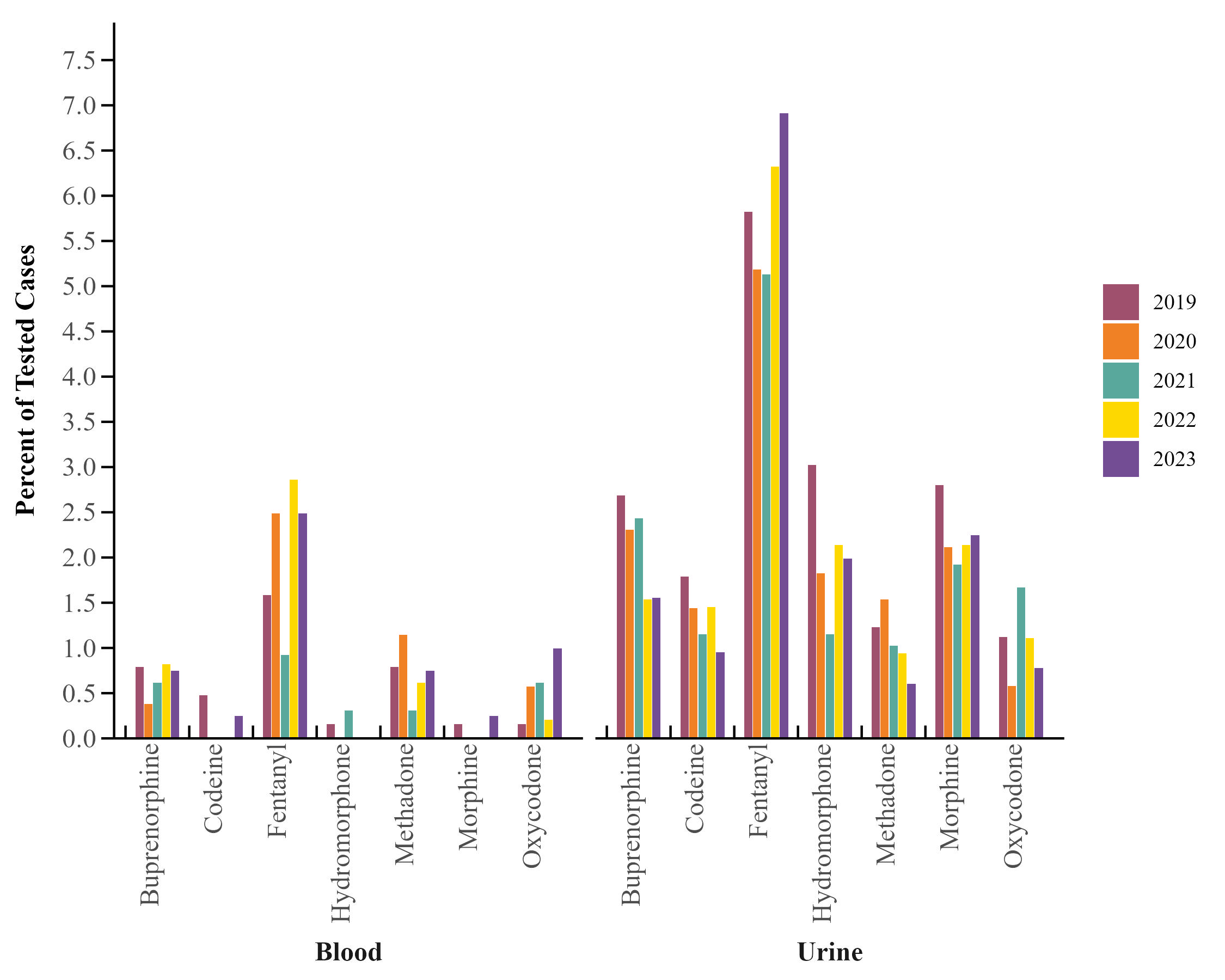

Supplement: Supplementary file 3 — Figure S2. Percent positivity by opioid analyte of for all samples tested in blood and urine by year. [file JFO-70-2442-s004.jpeg]

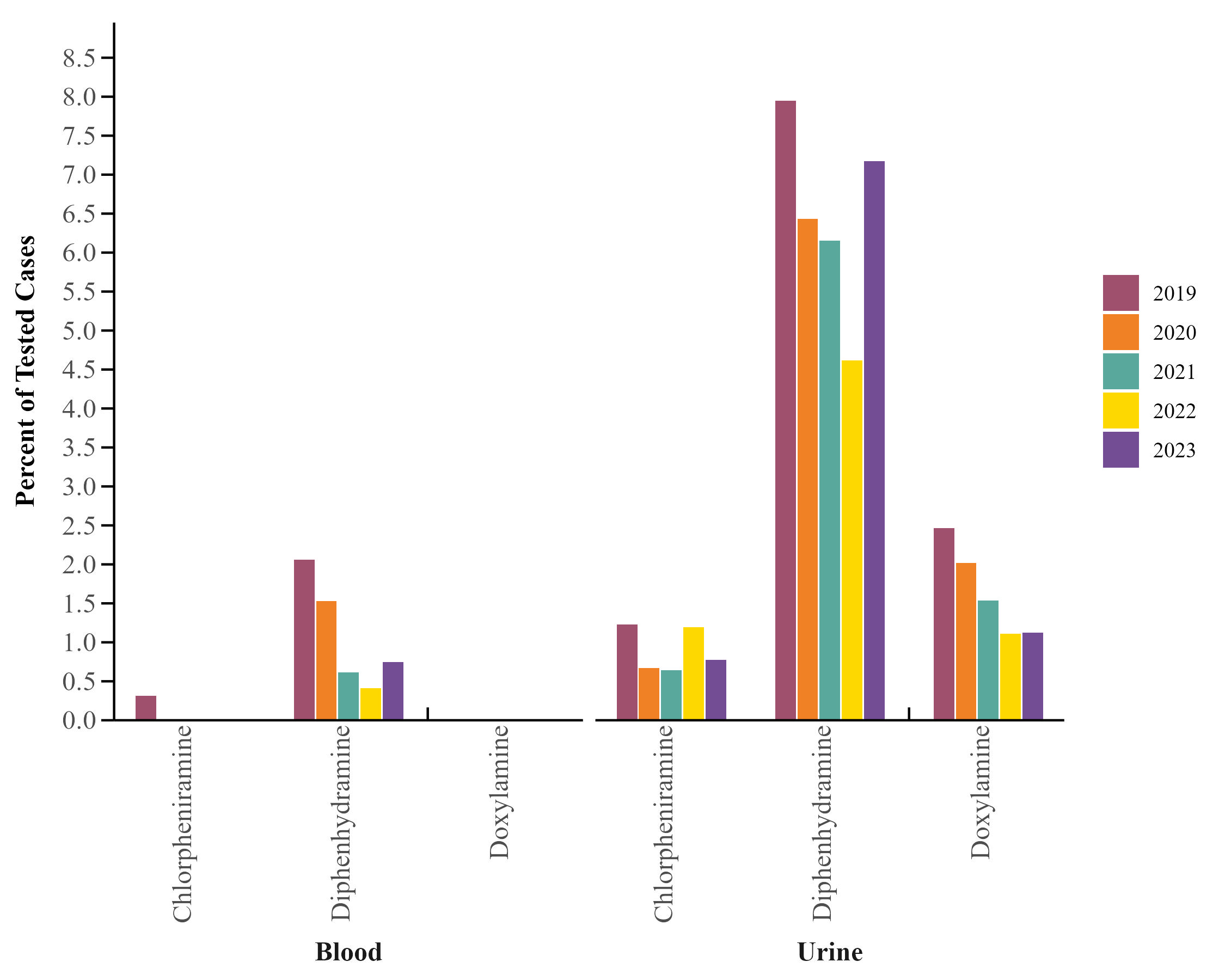

Supplement: Supplementary file 4 — Figure S3. Percent positivity by antihistamine analyte of for all samples tested in blood and urine by year. [file JFO-70-2442-s001.jpeg]
